# The Herpes Simplex Virus-1 Transactivator Infected Cell Protein-4 Drives VEGF-A Dependent Neovascularization

**DOI:** 10.1371/journal.ppat.1002278

**Published:** 2011-10-06

**Authors:** Todd Wuest, Min Zheng, Stacey Efstathiou, William P. Halford, Daniel J. J. Carr

**Affiliations:** 1 Department of Microbiology and Immunology, University of Oklahoma Health Sciences Center, Oklahoma City, Oklahoma, United States of America; 2 Department of Ophthalmology, University of Oklahoma Health Sciences Center, Oklahoma City, Oklahoma, United States of America; 3 Department of Pathology, University of Cambridge, Cambridge, United Kingdom; 4 Department of Microbiology and Immunology, Southern Illinois University School of Medicine, Springfield, Illinois, United States of America; University of Glasgow, United Kingdom

## Abstract

Herpes simplex virus-1 (HSV-1) causes lifelong infection affecting between 50 and 90% of the global population. In addition to causing dermal lesions, HSV-1 is a leading cause of blindness resulting from recurrent corneal infection. Corneal disease is characterized by loss of corneal immunologic privilege and extensive neovascularization driven by vascular endothelial growth factor-A (VEGF-A). In the current study, we identify HSV-1 infected cells as the dominant source of VEGF-A during acute infection, and VEGF-A transcription did not require TLR signaling or MAP kinase activation. Rather than being an innate response to the pathogen, VEGF-A transcription was directly activated by the HSV-1 encoded immediate early transcription factor, ICP4. ICP4 bound the proximal human VEGF-A promoter and was sufficient to promote transcription. Transcriptional activation also required cis GC-box elements common to the VEGF-A promoter and HSV-1 early genes. Our results suggest that the neovascularization characteristic of ocular HSV-1 disease is a direct result of HSV-1's major transcriptional regulator, ICP4, and similarities between the VEGF-A promoter and those of HSV-1 early genes.

## Author Summary

Herpes simplex virus-type 1 is the leading cause of infectious corneal blindness in the industrialized world. Most of the morbidity associated with the virus is due to the host response to episodic reactivation of latent virus. Corneal immunologic privilege is associated with a number of factors including the absence of blood and lymphatic vessels. Conversely, corneal hem (blood)- and lymph-angiogenesis driven by inflammation correlate with the loss of privilege. Neovascularization is a common phenomenon in HSV-1 keratitis that correlates with poor prognosis. We have previously discovered HSV-1 elicits corneal lymphangiogenesis through a unique mechanism involving vascular endothelial growth factor (VEGF)-A independent of that described for other insults including transplantation or bacterial infection. However, the viral-encoded product(s) that elicit host production of VEGF-A is(are) unknown. In this paper, we have identified infected cell protein-4 (ICP4) as the primary virus-encoded product that drives VEGF-A expression. As VEGF-A is involved in driving neovascularization associated with tumor growth and metastasis, proteins that influence transcriptional regulation of VEGF-A may be useful in the development of adjunct therapy for such disparate diseases as cancer and HSV-1 keratitis.

## Introduction

Herpes simplex virus-type 1 (HSV-1) is a neurotropic member of the alpha herpesvirus family with worldwide seroprevalence rates ranging from between 50–90%.[1], [2]. Primary infection is usually mild or asymptomatic in the immunocompetent host and typically occurs in childhood or early adolescence following inoculation of mucosal epithelial surfaces. During initial infection, virions gain access to sensory nerve fibers and are transported to neuronal cell bodies in the trigeminal ganglia where HSV-1 establishes a latent infection [3].

Although treatable, infection is life-long as a result of the sequestration of latent virus from immunological surveillance [3]. Latency may be broken during times of stress or immunological suppression resulting in the resumption of the lytic viral replication cycle. Newly produced virions migrate down trigeminal nerve fibers to epithelial surfaces where the reactivated virus resumes lytic viral replication and infectious virions are released. Symptoms of reactivation may be as mild as dermal vesicles or as severe as herpes simplex encephalitis, the most common cause of sporadic viral encephalitis in the world [4].

Despite the familiarity of dermal HSV-1 lesions, the most significant clinical consequence of HSV-1 infection is secondary to ocular HSV-1 infection. The trigeminal nerve provides sensation to the lips, nose, and eye. Although the skin about the orofacial region is the most frequent target of viral reactivation, all areas innervated by the trigeminal nerve branches are susceptible and recurrent bouts of corneal reactivation are not uncommon [3], [5]. Repeated incidents of corneal infection lead to the breakdown of corneal immunologic privilege and the development of an immunoinflammatory disorder termed herpetic stromal keratitis (HSK). Chronic inflammation elicits extensive corneal opacification driven by host CD4+ T cells and neovascularization secondary to disruption of the normal equilibrium between corneal angiogenic and anti-angiogenic factors [5]. The immunoinflammatory nature of HSK is particularly vexing, as patients refractory to treatment with antiviral medication may require corneal transplantation [5]. Inflammation and corneal vascularization promote corneal graft failure [5]. Thus, the HSK-associated inflammatory processes which necessitate corneal transplantation also substantially increase the risk of transplant rejection in HSK patients [5].

Several different mechanisms contribute to corneal immunologic privilege including the expression of immunosuppressive factors, specialized tolerance-promoting DC populations, and the avascular nature of the cornea [6]–[8]. Corneal avascularity may play an important role during HSK, as corneal neovascularization is highly predictive of future graft failure in HSV-1 affected patients [5]. Furthermore, avascular tissues are universally immunologically privileged [9]–[11]. The cytokine vascular endothelial growth factor-A (VEGF-A) plays a particularly crucial role in HSV-1-induced corneal neovascularization and drives both angiogenesis and lymphangiogenesis [9], [12]–[14]. A recent study in our laboratory revealed that VEGF-A is expressed by HSV-1 infected corneal epithelial cells due to increased accumulation of VEGF-A mRNA in HSV-1 infected cells [12]. However, the mechanism by which VEGF-A expression is induced remains unclear.

In the current study, we report the discovery that transcriptional up-regulation of VEGF-A is dependent on HSV-1's major transcriptional regulator, infected cell protein 4 (ICP4). ICP4 binds the proximal human VEGF-A promoter and is sufficient for transcriptional up-regulation of VEGF-A. Additionally, VEGF-A expression requires a tract of GC-rich sequences with high homology to the promoters of HSV-1 early (E) genes that ICP4 normally transactivates. Our results indicate that HSV-1 promotes expression of VEGF-A via ICP4-dependent transactivation, and this may be the result of sequence similarity between HSV-1 E genes and the human proximal VEGF-A promoter.

## Results

### Expression of VEGF-A In Vitro and In Vivo by HSV-1 Infected Cells

**Figure 1:**
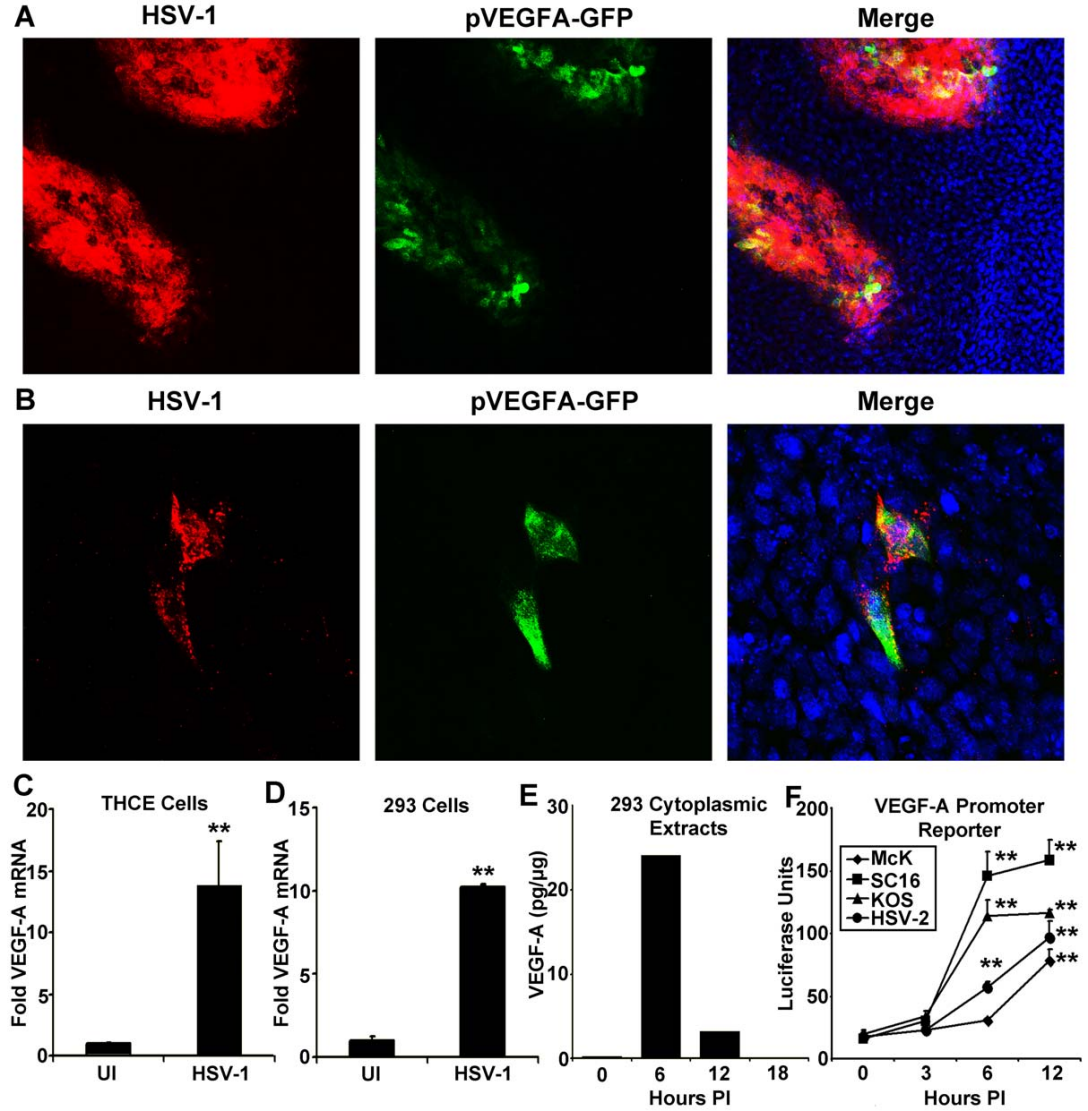
HSV-1 infection drives transcriptional upregulation of VEGF-A. (A) Representative image of a cornea from pVEGFA-GFP reporter mice at day 3 PI with HSV-1 strain McKrae stained for HSV-1 antigen (red), GFP transcriptional reporter for VEGF-A (green) and DAPI (blue) (B) GFP transcriptional reporter expression (green) with HSV-1 antigen (red) at 12 hours PI with HSV-1 strain McKrae. (C) Real time PCR of THCE cells at 12 hours PI with 3 pfu per cell HSV-1 strain McKrae with VEGF-A fold induction determined via the geometric means of VEGF-A induction relative to the housekeeping genes β-actin, TBP and PPIA (** p<0.01). (D) Real time PCR of VEGF-A transcription in 293 cells infected with 3 pfu per cell HSV-1 strain McKrae also determined via geometric mean relative to β-actin, TBP and PPIA (** p<0.01) and (E) Levels of VEGF-A by cytokine bead array in cytoplasmic extracts of 293 cells at the indicated times PI with 3 pfu per cell HSV-1 strain McKrae, expressed as pg of VEGF-A per ug of cytoplasmic protein extract. (F) Transcriptional activity of the proximal human VEGF-A promoter was measured using a luciferase vector with luciferase driven by the VEGF-A promoter (spanning base pairs −2018 to +50 bp relative to the transcription start site). Transfected 293 cells were infected with 3 pfu per cell of either wild-type HSV-1 strains; McKrae, KOS, or SC16 or with 3 pfu per cell HSV-2 and assayed for luciferase activity at the indicated time PI. Luciferase activity was normalized to the activity of uninfected 293 cells transfected with a promoterless luciferase vector, pGL3 (** p<0.01). All figures are representative figures from individual experiments with an n = 3/group for A–D. Extracts from 3 culture wells per time point were pooled for E. Bars denote mean VEGF-A pg per µg of cytoplasmic protein ± SEM.

The human cornea is normally devoid of blood and lymphatic vessels due to low expression of (lymph)angiogenic cytokines and abundant expression of anti-(lymph)angiogenic factors [6], [8], [10], [11]. Our group has previously described the expression of the pro-(lymph)angiogenic cytokine VEGF-A by HSV-1 infected corneal epithelial cells which drives lymphangiogenesis following HSV-1 infection [12]. Increased expression of VEGF-A was at least partially the result of transcriptional up-regulation as mice expressing GFP under the proximal human VEGF-A promoter showed selective induction of GFP within HSV-1 antigen-positive cells at 36 hours post infection (PI) with HSV-1 strain McKrae (Figure 1A). Likewise, GFP reporter expression was detectable by 12 hours PI (Figure 1B). Real time PCR analysis of VEGF-A mRNA relative to the housekeeping genes β-actin, TBP, and PPIA demonstrated transcriptional up-regulation following HSV-1 infection in Tert-immortalized human corneal epithelial cells (THCE) (Figure 1C, p<0.01). Likewise, HSV-1 infection of the human embryonic kidney fibroblast 293 cell line yielded a similar transcriptional up-regulation of VEGF-A in human cells (Figure 1D). VEGF-A was also detected in cytoplasmic extracts of 293 cells with the highest detectable concentration being found at 6 hours PI (Figure 1E). VEGF-A promoter activity was also assayed using a reporter vector driving firefly luciferase under the proximal human VEGF-A promoter. Infection of 293 cells with three different wild-type HSV-1 strains (-McKrae, KOS, and SC16), and the homologous HSV-2 virus, all significantly induced expression of the VEGF-A promoter luciferase reporter gene (Figure 1F). Reporter expression peaked at 12 hours PI, which was 6 hours after VEGF-A levels peaked in the cytoplasm, possibly due to the differential secretion of VEGF-A versus the non-secreted luciferase reporter which was retained in cells. Thus, HSV-1 infection drove transcriptional up-regulation of VEGF-A and protein expression both *in vitro* and *in vivo*.

**Figure 2:**
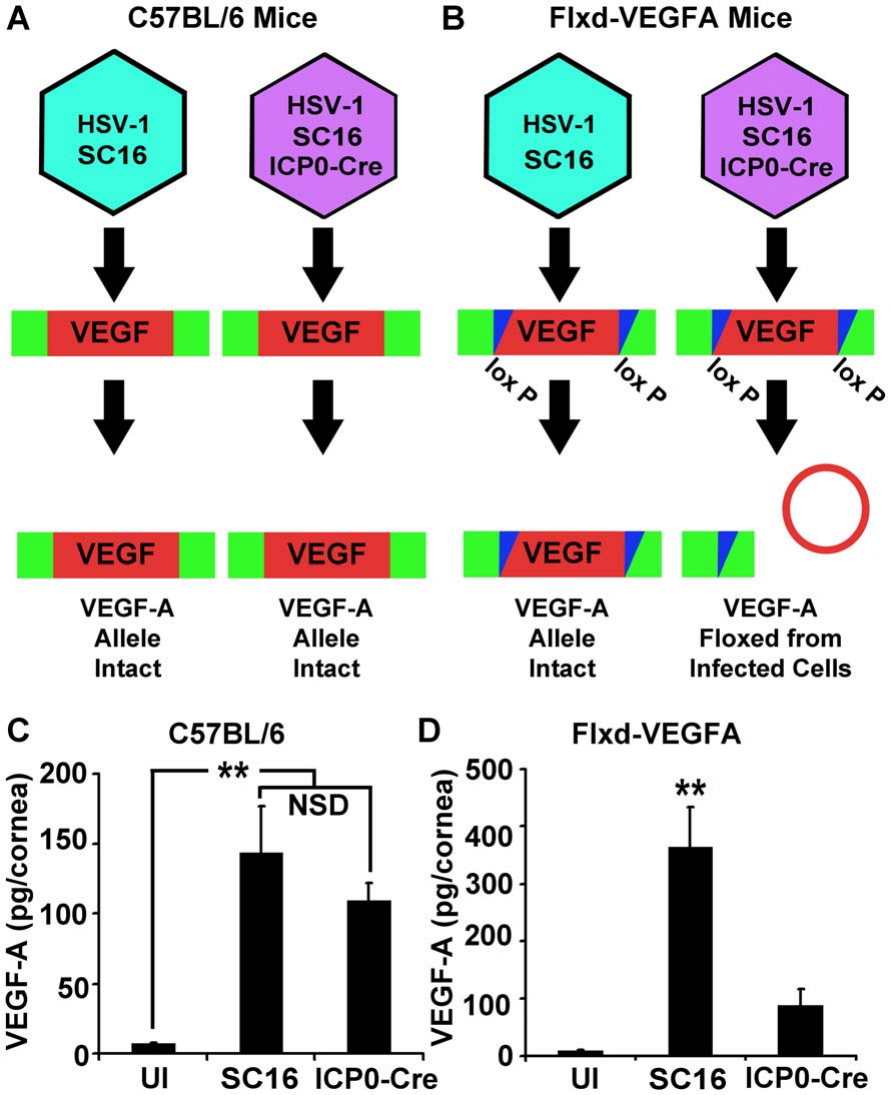
HSV-1 infected cells are the dominant source of VEGF-A during acute HSV-1 infection. To assess the relative contribution of HSV-1 infected or uninfected cells to global VEGF-A expression, C57BL/6 (A) or Flxd-VEGFA (B) mice were infected with either wild-type HSV-1 SC16 or a derivative expressing Cre under the ICP0 promoter sequence resulting in the selective Cre-mediated ablation of VEGF-A expression in ICP0-Cre infected Flxd-VEGFA cells. (C) VEGF-A levels in the corneas of wild-type C57BL/6 control animals which were scarified only (UI) or scarified and infected with 10^5^ pfu of HSV-1 strain SC16 or SC16 expressing Cre under the ICP0 promoter (ICP0-Cre) and harvested at 24 hours PI, (** p<0.01, NSD non-significant difference). (D) VEGF-A levels were not elevated in the corneas of animals in which the VEGF-A allele was floxed after receiving ICP0-Cre relative to UI controls. However, VEGF-A was up-regulated in animals received HSV-1 strain SC16 at 24 hours PI. (** p<0.01). Figures A and B are summaries of 2 experiments with 2 corneas per sample, total n = 4 samples in UI group and a total n = 5 samples in each infected group. Bars denote mean VEGF-A pg/mg of cornea wet mass ± SEM.

VEGF promoter-driven GFP expression was only detected in HSV-1 antigen-positive cells within the cornea during acute infection [12]. However, HSV-1 antigen-negative cells expressing GFP reporter were observed in limbal tissues, proximal to the cornea (data not shown). Neutrophils contain preformed stores of VEGF-A and could release VEGF-A during HSV-1 infection without detectably expressing reporter for VEGF-A transcription [15]. To determine the relative contribution of HSV-1 infected versus uninfected cells to VEGF-A production *in vivo*, we utilized the Cre-lox system to selectively excise the VEGF-A gene from HSV-1 infected cells. Either C57BL/6 controls or mice with a floxed VEGF-A gene (Flxd-VEGFA) were infected with either wild type HSV-1 strain SC16 or a Cre-expressing HSV-1 recombinant virus, which was derived from strain SC16 and expressed Cre under the control of the HSV-1 ICP0 promoter (Figure 2A and B). In C57BL/6 control mice, corneal VEGF-A concentrations did not significantly differ at day 3 PI between mice inoculated with wild type HSV-1 versus the Cre-expressing HSV-1 recombinant (Figure 2C, p>0.05). However, in Flxd-VEGFA mice, VEGF-A concentrations at day 3 PI were 75% lower in animals infected with the Cre-expressing ICP0 recombinant virus relative to Flxd-VEGFA miceinfected with wild type HSV-1 (Figure 2D). Based on the fact that selective Cre-mediated excision of the VEGF-A gene reduced VEGF-A levels by 4-fold, it appeared that HSV-1 infected cells were the predominant source of VEGF-A during acute HSV-1 infection.

### VEGF-A Expression Was Not Dependent on the Innate Pattern Recognition Receptor Adaptor Proteins MyD88 or TRIF

**Figure 3:**
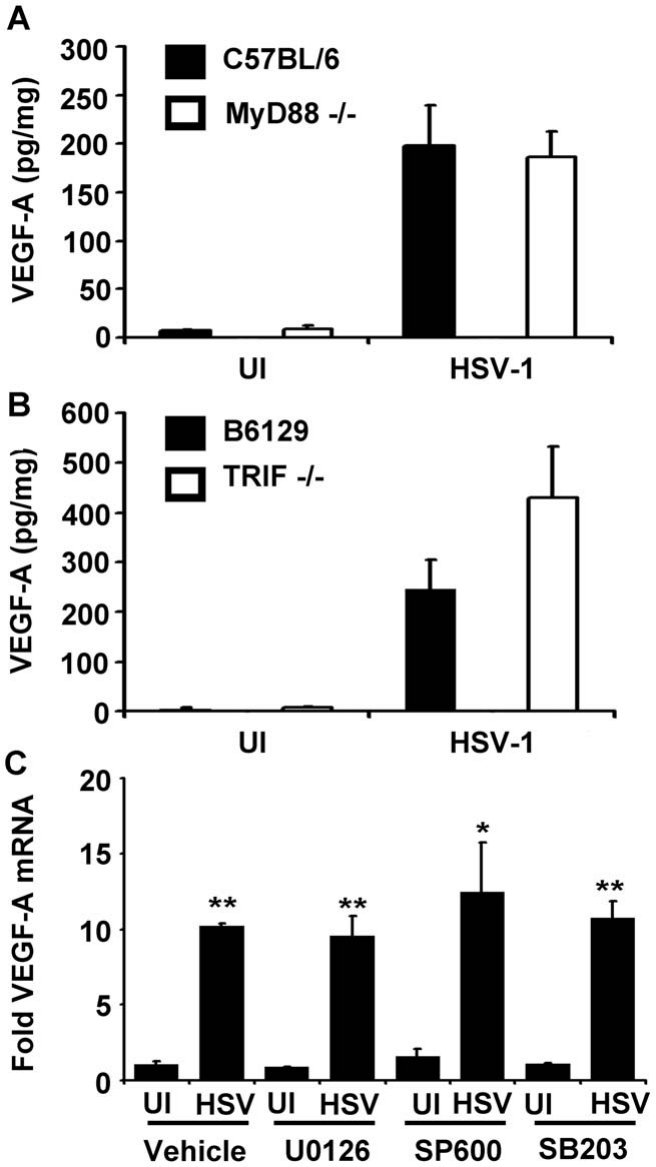
VEGF-A expression is not dependent on the TLR adaptors MyD88 or TRIF. VEGF-A levels expressed as pg of VEGF-A per mg of cornea wet mass at 24 hours after scarification alone (UI) or infection with 10^5^ pfu of HSV-1 strain McKrae in either (A) C57BL/6 controls or MyD88−/− animals or (B) B6129 controls or TRIF−/− animals. (C) Human 293 cells were plated and fold induction of VEGF-A transcript after infection with 3 pfu per cell HSV-1 strain McKrae. Treatment with inhibitors of MEK1/2 (U0126), JNK1/2 (SP600125) or p38 (SB206580) at 10 µM concentrations also did not block VEGF-A transcription relative to uninfected, vehicle-treated control. Bars denote ± SEM. A and B are summaries of two experiments with total n = 6/group. Panel C is a representative figure of 2 experiments, n = 3/group/experiment. Fold induction values were normalized to VEGF-A levels in uninfected, vehicle-treated controls using the geometric mean of fold induction values relative to the housekeeping genes β–actin, TBP, and PPIA. (** p<0.01, *<0.05, NSD non-significant difference).

VEGF-A is expressed during a wide range of inflammatory processes including wound healing, psoriasis, and following the ligation of some toll-like receptors (TLR) [16], [17]. HSV-1 infection stimulates the TLRs 2, 3, 4, and 9 [18]. All TLRs depend on the MyD88 and/or TRIF adaptor proteins for signal transduction [19]. Therefore, we tested whether VEGF-A was TLR-dependent using mice deficient in MyD88 (MyD88^−/−^) or TRIF (TRIF^−/−^). Corneas of MyD88^−/−^ mice and TRIF^−/−^ or their respective C57BL/6 and B6129 controls strains, were scarified and inoculated with PBS or 10^5^ PFU of HSV-1. Corneas were harvested at 24 hours PI and assayed for VEGF-A levels by cytokine bead array. HSV-1 infection induced VEGF-A expression to high and statistically equivalent levels in the corneas of C57BL/6, MyD88^−/−^, B6129, and TRIF^−/−^ mice (Figure 3A and B). While VEGF-A levels were slightly higher in TRIF^−/−^ mice relative to B6129 controls, this difference was not significant (Figure 3B, p>0.05). In addition, VEGF-A dependent corneal lymphangiogenesis was equivalent in MyD88^−/−^ and TRIF^−/−^ mice at day 5 PI relative to wild type control mice (data not shown).

MAP kinase activation up-regulates VEGF-A expression in tumors and following TLR ligation [20]–[22]. Activation of MEK following *Helicobacter pylori* infection induces VEGF-A expression through the transcription factors Sp1 and Sp3 [23]. The HSV-1 immediate early (IE) gene product, ICP27 activates JNK and p38 pathways [24], and the HSV-2 homolog of the HSV-1-encoded protein kinase, US3, directly activates the MEK pathway [25]. Therefore, we sought to clarify if any of these signal transuction pathways might play a role in HSV-1 stimulated VEGF-A expression. Using 293 cells, we tested if HSV-1 induction of VEGF-A mRNA accumulation was dependent on MEK1/2, p38, or JNK1/2 signal transduction pathways using their respective inhibitors, U1026, SB206580, and SP600125 (Figure 3C). Treatment of cells with 10 µM of each inhibitor (≥100 times the IC_50_ values of each drug) had no discernable effect on the up-regulation of VEGF-A mRNA levels 12 hours PI relative to vehicle- treated cells (Figure 3C). Therefore, neither MyD88-, TRIF-, MAP kinase-, MEK1/2-, p38-, or JNK1/2-signal transduction pathways appeared to be required for HSV-1 infection to induce VEGF-A accumulation in HSV-1 infected cells.

### VEGF-A Promoter Analysis

**Figure 4:**
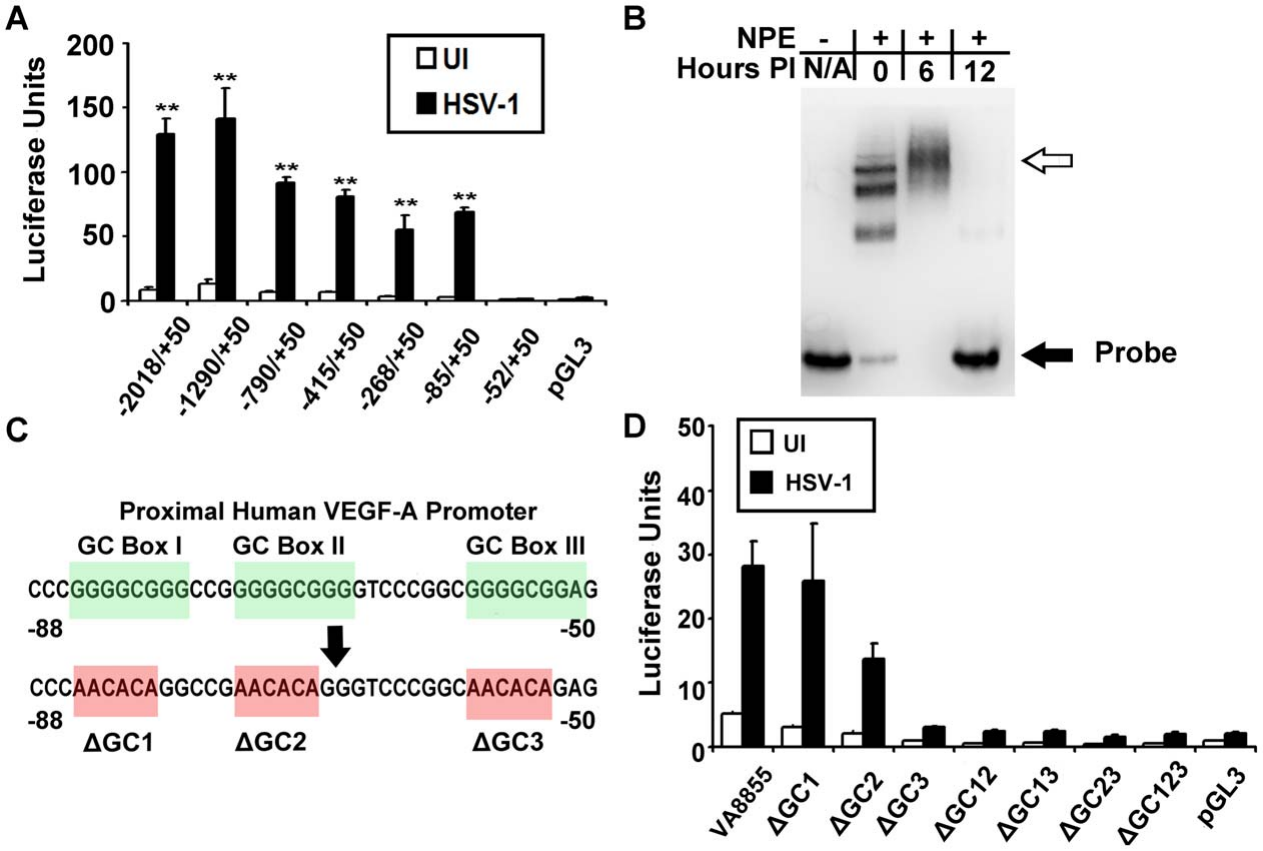
VEGF-A promoter analysis. (A) Human 293 cells were transfected with luciferase reporter plasmids (pGL3) driven by the indicated segments of the human VEGF-A promoter relative to the transcription start site. Luciferase activity was measured at 12 hours PI with 3 pfu per cell HSV-1 strain McKrae. HSV-1 induced upregulation of luciferase activity required a GC-rich segment spanning base pairs −85 to −52 diagramed in (C). (B) EMSA showing differential binding of nuclear proteins to VEGF-A −88 to +55 base pair probe. Free probe denoted by the solid black arrow was shifted following addition of nuclear extracts from uninfected 293 cells and probe mobility was further retarded by nuclear proteins harvested from cells at 6 hours PI with 3 pfu per cell HSV-1 (hollow arrow). By 12 hours PI, probe mobility was no longer shifted by nuclear extracts. (C) Diagram of the proximal human VEGF-A promoter showing “GC box” consensus site for transcription factors of the Sp1 family. Bases shown in red indicate base changes to render a luciferase construct driven by base pairs −88 to +55 of the human VEGF-A promoter to being devoid of individual or multiple GC box sequences. (D) Luciferase activity was measured in 293 cells transfected with either wild-type pVA8855 reporter plasmid or the indicated GC box mutations at 12 hours PI with 3 pfu per cell HSV-1 strain McKrae. Representative figure from 2 experiments, n = 3/group/experiment.

To clarify which promoter elements were required for HSV-1 to induce expression from the VEGF-A promoter, we utilized luciferase reporter plasmids containing human VEGF-A promoters that ranged in size from 2068 base pair (bp) promoter that spanned −2018 to +50 bp (relative to the transcription start site) to a minimal 102-bp VEGF-A promoter that spanned −52 to +50 bp (Figure 4A). Transfected 293 cells were then assayed for luciferase expression at 12 hours PI with HSV-1 strain McKrae (Figure 4A). Expression depended on a short stretch of DNA from −85 to −52 bp relative to the transcription start site. The absence of HIF-1α elements (−975 to −968 bp) or STAT3 elements (−848 to −840 bp), which mediate VEGF-A transcriptional up-regulation in response to hypoxia and IL-6 respectively [25] did not prevent HSV-1 from inducing a 10-fold increase in luciferase expression from the −790 to +50 BP promoter (Figure 4A).

EMSA analysis also demonstrated a distinct alteration in the binding of nuclear proteins to biotinylated probe spanning −88 to +55 bp following infection with HSV-1. In uninfected 293 cells, nuclear protein extracts bound probe in discrete shifts, possibly corresponding to either multimeric structures or distinct complexes (Figure 4B). In contrast, nuclear protein extracts harvested 6 hours after HSV-1 McKrae infection contained proteins that primarily bund the VEGF-A promoter probe in a in a broad band of lower mobility. The same probe was not bound to detectable levels by nuclear extracts harvested from HSV-1 strain McKrae-infected cells at 12 hours PI (Figure 4B). However, during infection with the less virulent strain HSV-1 strain KOS, an EMSA shift was still detectable at 12 hours PI (data not shown).

The original construction of the luciferase reporter plasmid driven by the −85 to 50 bp segment of the human VEGF-A promoter resulted in the loss of the pGL3 multiple cloning site (MCS). A new and equivalent luciferase expression vector was constructed that retained a MCS, and thus allowed site-directed mutagenesis of the proximal VEGF-A promoter from −88 to +55 bp relative to the transcription start site. The resulting plasmid was denoted [pVA8855] and performed equivalently to the original plasmid vector. Three consensus “GC box” sequences are present between 85 to −52 bp of the VEGF-A promoter, and which may serve as binding sites for transcription factors of the Sp family [22] (Figure 4C).

We tested the relevance of these GC box sequences in the VEGF-A promoter by mutating individual GGGCGG consensus sequences, or combinations thereof, to the mutant sequence AACACA (Figure 4C). Human 293 cells were transiently transfected with the wild type VEGF-A promoter construct, pVA8855, or 1 of 8 GC-box mutant plasmids. After 48 hours, cells were mock-inoculated or inoculated with 3 pfu per cell of HSV-1 strain McKrae, and luciferase levels were compared at 12 hours PI (Figure 4D). Mutation of GC-box 1 alone (−85 to −80 bp) or GC-box 2 alone (−74 to −69 bp) did not preclude HSV-1 induction of the luciferase reporter gene (Figure 4D). In contrast, deletion of GC-box 3 (−58 to −53 bp) or any permutation of two or more GC-boxes ablated the capacity of HSV-1 to induce luciferase expression from the minimal VEGF-A promoter represented by −88 to +50 bp relative to the transcriptional start site (Figure 4D).

Although GC box sequences were required for VEGF-A promoter transcriptional up-regulation, we did not detect binding of nuclear proteins to GC boxes using proximal VEGF-A promoter probes. EMSA assays of native or GC box mutated probes (Figure S1A) spanning −88 to −50 bp relative to the transcription start site did not show differential EMSA shifts between probes using either uninfected or 6 hour PI nuclear extracts (Figure S1B). However, we presume this negative result was due to extensive secondary structure within this region of the human VEGF-A promoter blocking transcription factor binding as probe containing an isolated GC box probe was shifted using nuclear extracts from 0, 6, and 12 hours PI (Figure S1C).

### No Apparent Role for EGR-1

**Figure 5:**
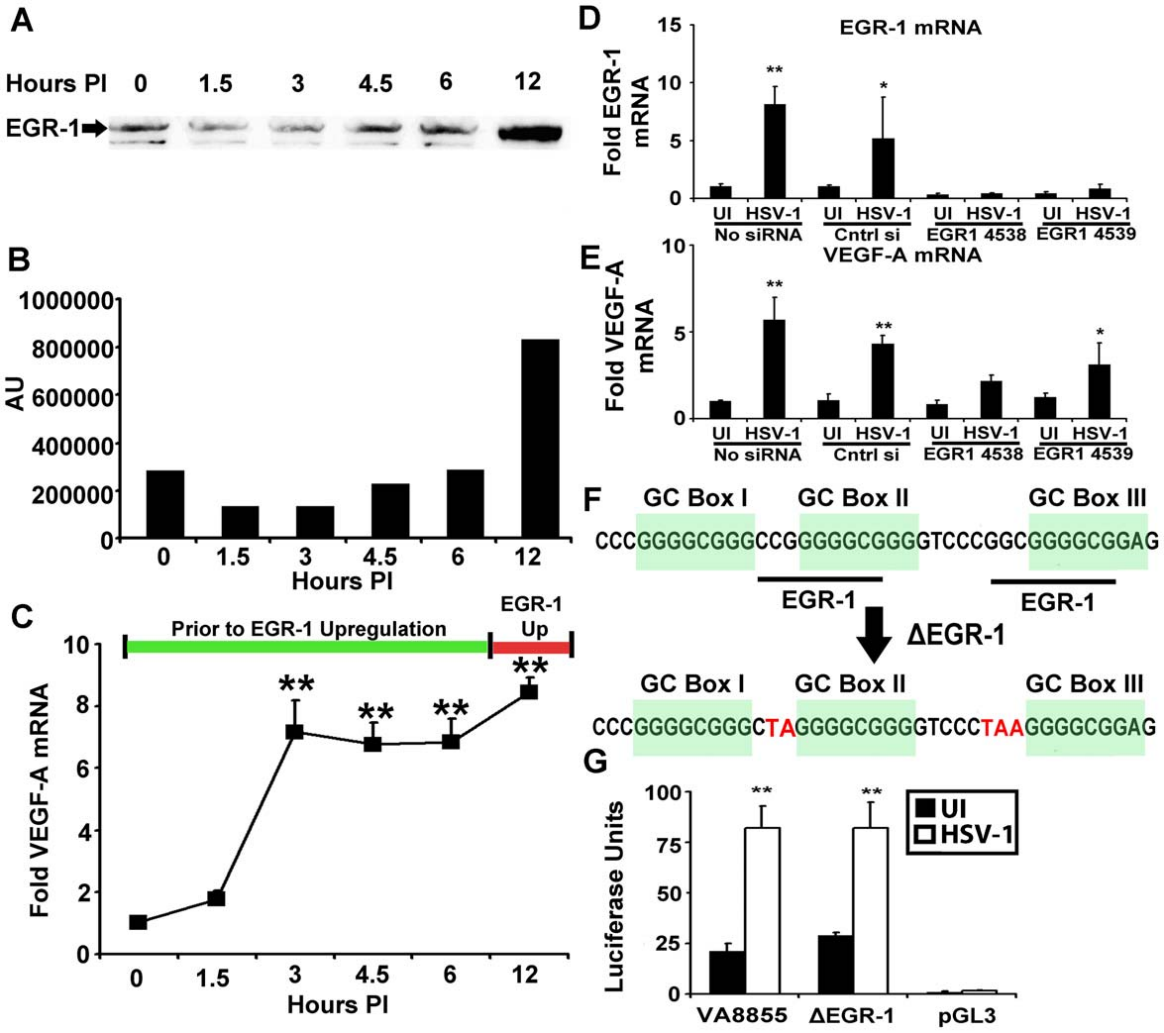
EGR-1 is not required for VEGF-A Upregulation. (A) Human 293 cells were infected with 3 pfu per cell HSV-1 McKrae, and nuclear protein extracts harvested at the indicated time PI were assayed for EGR-1 by western blot. (B) Densitometry values of EGR-1 western blots at the indicated time PI. (C) Fold induction of VEGF-A mRNA relative to the β-actin, TBP and PPIA at the indicated time PI with 293 cells infected with 3 pfu per cell HSV-1 McKrae. (D) EGR-1 mRNA fold induction relative to β-actin, TBP and PPIA following HSV-1 infection in 293 cells treated with either vehicle, negative control siRNA, or two independent siRNAs against EGR-1 (4538 and 4539). Individual fold induction values were determined via geometric means of fold induction of VEGF-A transcript relative to the housekeeping genes β-actin, TBP and PPIA. Reduction relative to respective uninfected controls was approximately 60%. The EGR-1 transcript was not up-regulated following HSV-1 infection in siRNA treated groups. Comparison between HSV-1 infected non-transfected cells versus HSV-1 infected cells treated with siRNA 4539 for EGR-1 transcript showed a reduction of approximately 89% and greater than 95% reduction for cells treated with siRNA 4538 relative to non-transfected, HSV-1 infected cells, p<0.01. (**p<0.01, * p<0.05). (E) VEGF-A fold induction following siRNA treatment. Transcriptional up-regulation occurred in the absence EGR-1 up-regulation in 4539 treated cells and tended toward up-regulation in 4538 treated cells as well. (F) Diagram of the human VEGF-A promoter from base pairs −88 to −50 showing GC box and EGR-1 consensus sequences along with mutations introduced to obviate EGR-1 binding to reporter plasmid shown in red (ΔEGR-1). (G) Luciferase activity of 293 cells transfected with either control VEGF-A −88 to +55 (pVA8855) base pair promoter driven reporter construct, ΔEGR-1 reporter, or the promoterless control reporter backbone pGL3, after infection with 3 pfu per cell HSV-1 strain McKrae. (** p<0.01). (** p<0.01, * p<0.05). Figures are representative of 2 experiments, C–E and F used an n = 3/group/experiment. Bars denote arithmetic mean of individual fold induction values ± SEM.

HSV-1 infection up-regulates the transcription factor EGR-1 which promotes transcription at sites within the HSV-1 genome bearing the core consensus sequence GCGGGGGCG [26]. The region of the human VEGF-A promoter spanning −85 to −52 bp contains two EGR-1 binding sequences, and EGR-1 drives expression of VEGF-A following growth factor stimulation [22]. Western blot analysis of HSV-1 infected 293 cells for EGR-1 did not demonstrate up-regulated expression of EGR-1 until 12 hours PI (Figure 5A and B). In contrast, VEGF-A transcript was significantly augmented (p<.01) as early as 3 hours PI suggesting that an EGR-1 independent pathway activated the VEGF-A promoter in HSV-1 infected cells (Figure 5C).

To test for the possibility of either an indirect or direct role for EGR-1, we used siRNA to reduce EGR-1. Transfection with siRNAs against EGR-1 (4538 and 4539) blocked EGR-1 up-regulation following HSV-1 infection with EGR-1 expression reduced by roughly 89% (comparison between HSV-1 infected non-transfected cells versus HSV-1 infected cells treated with siRNA 4539, p<0.01, Figure 5D). However, even in the absence of EGR-1 up-regulation VEGF-A transcript expression was up-regulated following HSV-1 infection in siRNA 4539-treated cells and tended to increase following siRNA 4538 treatment as well (Figure 5E).

EGR-1 is constitutively expressed in 293 cells, and EGR-1 affects HSV-1 gene expression [26]. Thus, EGR-1 knockdown may indirectly affect pathways that could influence VEGF-A transcription. We tested for a direct role for EGR-1 via site specific mutagenesis of EGR-1 binding sequences within the VEGF-A promoter. EGR-1 is a C2H2-type three zinc finger transcription factor with the fingers binding the triplets GCG, G/T GG, and GCG respectively [27]. EGR-1 and GC box elements within the −85 to −52 bp region of the VEGF-A promoter overlap so only modification to the first triplet of EGR-1 sites could be made without also altering GC box sequences (Figure 5F). In fact, mutation of the first triplet is sufficient to abrogate EGR-1 binding [28], [29]. Mutation of EGR-1 consensus sequences had no effect on VEGF-A promoter driven luciferase expression following HSV-1 infection (pVA8855 versus ΔEGR-1 or promoterless reporter vector pGL3, Figure 5G). Although EGR-1 could conceivably contribute to transcription of the VEGF-A gene at late stages of HSV-1 infection, we did not observe a requirement for either EGR-1 up-regulation or the presence of EGR-1 consensus sites for transcriptional up-regulation of VEGF-A.

### HSV-1 ICP4 Is Required for VEGF-A Transcription and Reporter Expression

**Figure 6:**
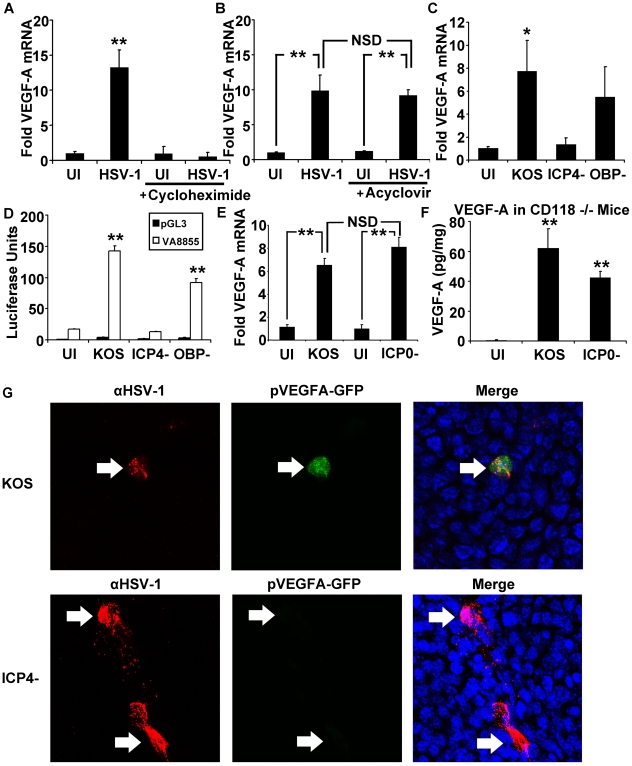
The HSV-1 transactivator ICP4 is required for VEGF-A expression. Human 293 cells were plated and infected with 3 pfu per cell HSV-1 strain McKrae. Fold induction of VEGF-A transcript expression was determined via geometric means of fold induction of VEGF-A transcript relative to the housekeeping genes β-actin, TBP and PPIA. (A) Inhibition of protein synthesis with cycloheximide (100 µg/mL) blocked upregulation of VEGF-A following HSV-1 infection while (B) inhibition of viral DNA synthesis with acyclovir (200 µM) had no significant effect. (C) ICP4 dependence was tested using human 293 cells infected with 3 pfu per cell of wild type HSV-1 KOS or KOS-derived mutants lacking ICP4 (ICP4^−^ null virus) or the HSV-1 origin binding protein (OBP^−^ null virus). VEGF-A transcript was measured via real time RT-PCR relative to β-actin, TBP and PPIA. (*p<0.05 relative to uninfected control cells). (D) Human 293 cells transfected with pVA8855 or the promoterless luciferase control plasmid, pGL3. Luciferase activity was measured at 12 hours PI with HSV-1 KOS, ICP4^−^ null, or OBP^−^ null virus. (E) VEGF-A mRNA levels in human 293 cells infected with 3 pfu per cell HSV-1KOS or an HSV-1 ICP0^−^ null virus that fails to make the IE co-transactivator protein, ICP0; both viruses significantly up-regulated VEGF-A mRNA levels. (F) Mice lacking type I interferon responses due to deficiency in the type I interferon receptor (CD118^−/−^) were infected with HSV-1 KOS or HSV-1 ICP0^−^ null virus. Corneas were harvested at 24 hours PI with 10^5^ pfu of HSV-1 and analyzed for VEGF-A levels by cytokine bead array, expressed as pg of VEGF-A per mg of cornea wet mass. VEGF-A was induced by inoculation with HSV-1 KOS or HSV-1 ICP0^−^ null virus (** p<0.01 relative to uninfected, scarified controls). (G) Reporter mice expressing GFP under the human VEGF-A promoter were analyzed for HSV-1 antigen (red) and GFP (green) along with DAPI (blue) at 12 hours PI with either HSV-1 KOS or HSV-1 ICP4- virus. A and B are representative figures of two experiments, n = 3/group/experiment. Fold induction values were normalized to VEGF-A levels in uninfected, vehicle-treated controls. Panels C and D are representative of two experiments, n = 3/group/experiment. Panel E is a summary of two experiments, n = 6/group. Panel F is representative of two experiments with an n = 3/group/experiment. Bars denote mean ± SEM. (** p<0.01, *<0.05, NSD = non-significant difference).

Two experiments were conducted with biochemical inhibitors to determine if HSV-1's capacity to induce the VEGF-A promoter was dependent upon the *de* novo synthesis of viral or cellular proteins in HSV-1 infected cells. The first experiment used the protein synthesis inhibitor cycloheximide. When protein translation was allowed to occur, 3 pfu per cell of HSV-1 McKrae induced a 10-fold increase in VEGF-A mRNA levels in THCE cells at 12 hours PI (Figure 6A). A second experiment was conducted with the guanosine analogue acyclovir to determine if restriction of HSV-1 DNA synthesis and ∼445 HSV-1 late (L) proteins affected HSV-1's capacity to induced VEGF-A synthesis [30], [31]. Infection with HSV-1 McKrae induced VEGF-A mRNA to high and equivalent levels in THCE cells treated with vehicle or 200 µM acyclovir (Figure 6B). These data excluded the possibility that HSV-1 virion attachment and/or entry were sufficient to induce VEGF-A mRNA accumulation. Rather, the data suggested that *de* novo protein synthesis during the initial hours of HSV-1 infection was necessary to induce VEGF-A gene expression in HSV-1 infected cells.

To explore the possibility that one or more HSV-1 proteins might contribute to VEGF-A transcriptional up-regulation, the capacity of wild type HSV-1 strain KOS to induce VEGF-A mRNA accumulation was compared to two well characterized HSV-1 KOS-derived mutants, HSV-1 n12 [32] and HSV-1 hr94 [33]. HSV-1 n12 is an ICP4^−^ null virus that fails to encode HSV-1's major transcriptional regulator, infected cell protein 4 (ICP4), and consequently fails to exit the IE phase of protein accumulation [32]. As a result, HSV-1 ICP4^−^ null viruses over express four IE proteins (ICP0, ICP22, ICP27, and ICP47) and fail to efficiently synthesize the other ∼70 E and L proteins encoded by the HSV-1 genome. The second HSV-1 mutant, hr94, fails to encode HSV-1 origin-binding protein (OBP), which is necessary for the onset of viral DNA synthesis [33]. As a result, HSV-1 OBP^−^ null viruses efficiently synthesize ∼30 IE and E proteins, but exhibit more restricted expression of ∼45 HSV-1 L proteins.

The efficiency of VEGF-A mRNA induction was compared in 293 cells inoculated with 3 pfu per cell of wild type HSV-1 KOS versus the KOS-derived ICP4^−^ null (n12) or OBP^−^ null (hr94) viruses. HSV-1 KOS induced an average 8-fold increase in VEGF-A mRNA levels at 12 hours PI (Figure 6C). Likewise, the HSV-1 OBP^−^ null virus induced an average 6-fold increase in VEGF-A mRNA levels (Figure 6C). In contrast, infection with an HSV-1 ICP4^−^ null virus did not detectably induce VEGF-A mRNA accumulation (Figure 6C).

An experiment was conducted to determine if ICP4 played a role in the capacity of HSV-1 to induce the minimal VEGF-A promoter represented by −88 to +55 bp in luciferase reporter plasmid construct pVA8855 (Figure 4D). Specifically, 293 cells were transfected with pVA8855 or a promoterless control plasmid, pGL3, for 48 hours, and then the cells were infected with 3 pfu per cell of wild type, ICP4^−^ null, or OBP^−^ null HSV-1. Luciferase reporter gene expression was significantly elevated in 293 cells inoculated with HSV-1 KOS or the HSV-1 OBP^−^ null virus (Figure 6D, **p<.01). In contrast, inoculation with the HSV-1 ICP4^−^ null virus failed to induce luciferase expression from the VEGF-A promoter in pVA8855 (Figure 6D). Tests were conducted to determine if the IE transactivator ICP0 was necessary for HSV-1 to induce VEGF-A mRNA accumulation. Human 293 cells infected with an HSV-1 ICP0^−^ null virus, 7134, transcribed VEGF-A mRNA at levels equivalent to cells infected with the parental virus HSV-1 KOS (Figure 6E). Likewise, HSV-1 KOS and HSV-1 ICP0^−^ null virus were compared for their ability to induce VEGF-A production *in vivo* in the corneas of interferon-signaling deficient CD118^−/−^ mice, in which HSV-1 ICP0^−^ null viruses may replicate to nearly wild type levels during the first 24 hours PI [34]. In these *in vivo* tests, VEGF-A levels were significantly up-regulated at 24 hours PI in the corneas of mice inoculated with wild type HSV-1 KOS or the ICP0^−^ null virus relative to mock-infected controls (Figure 6F, **p<.01). Thus, ICP0 is not essential for HSV-1 infection to induce VEGF-A mRNA or protein accumulation.

We verified these conclusions in an *in vivo* test using pVEGFA-GFP reporter mice. Following inoculation of mouse corneas with HSV-1 ICP4^−^ null virus, HSV-1 antigen positive cells were detectable in the cornea at 12 hours PI, but these sites did not co-localize with induction of the pVEGFA-GFP reporter in these transgenic mice (Figure 6G). In contrast, mouse corneas inoculated with wild type KOS (Figure 6G) or HSV-1 OBP^−^ null virus (data not shown) exhibited a clear co-localization of HSV-1 antigen-positive cells and induction of the pVEGFA-GFP reporter gene. Although low level GFP expression was observed in HSV-1 antigen-positive cells in reporter mice infected with ICP4^−^ null virus, expression was extremely faint compared to that observed in KOS infected cells and was comparable to the transient GFP expression observed following corneal scarification procedure required for HSV-1 infection.

As an additional control we tested the ability of an HSV-1 ICP27^−^ null virus, d27-1 [35], to induce the minimal VEGF-A promoter in plasmid pV8855. ICP27, like ICP4, is a viral IE protein that is required for the efficient expression of E and L proteins [36]. Hence, HSV-1 ICP27^−^ null viruses over express viral IE proteins including ICP4 (Figure S3A and B), but fail to express most of other ∼70 E and L HSV-1 proteins [35]. Consistent with the observed over expression of ICP4, cells inoculated with an HSV-1 ICP27^−^ virus induced the VEGF-A promoter in pV8855 to express 7-fold and 60-fold higher levels of luciferase than was observed in cells infected with wild type HSV-1 or an HSV-1 ICP4^−^ virus, respectively (Figure S3C). This correlation between ICP4 over expression and elevated luciferase reporter induction in ICP27^−^ virus-infected cells suggested that the major transcriptional regulator of HSV-1, ICP4, might play a direct role in transcriptional induction of the VEGF-A promoter rather than acting via an indirect mechanism that required the synthesis of HSV-1 E or L proteins.

### HSV-1 ICP4 Binds the Proximal VEGF-A Promoter

**Figure 7:**
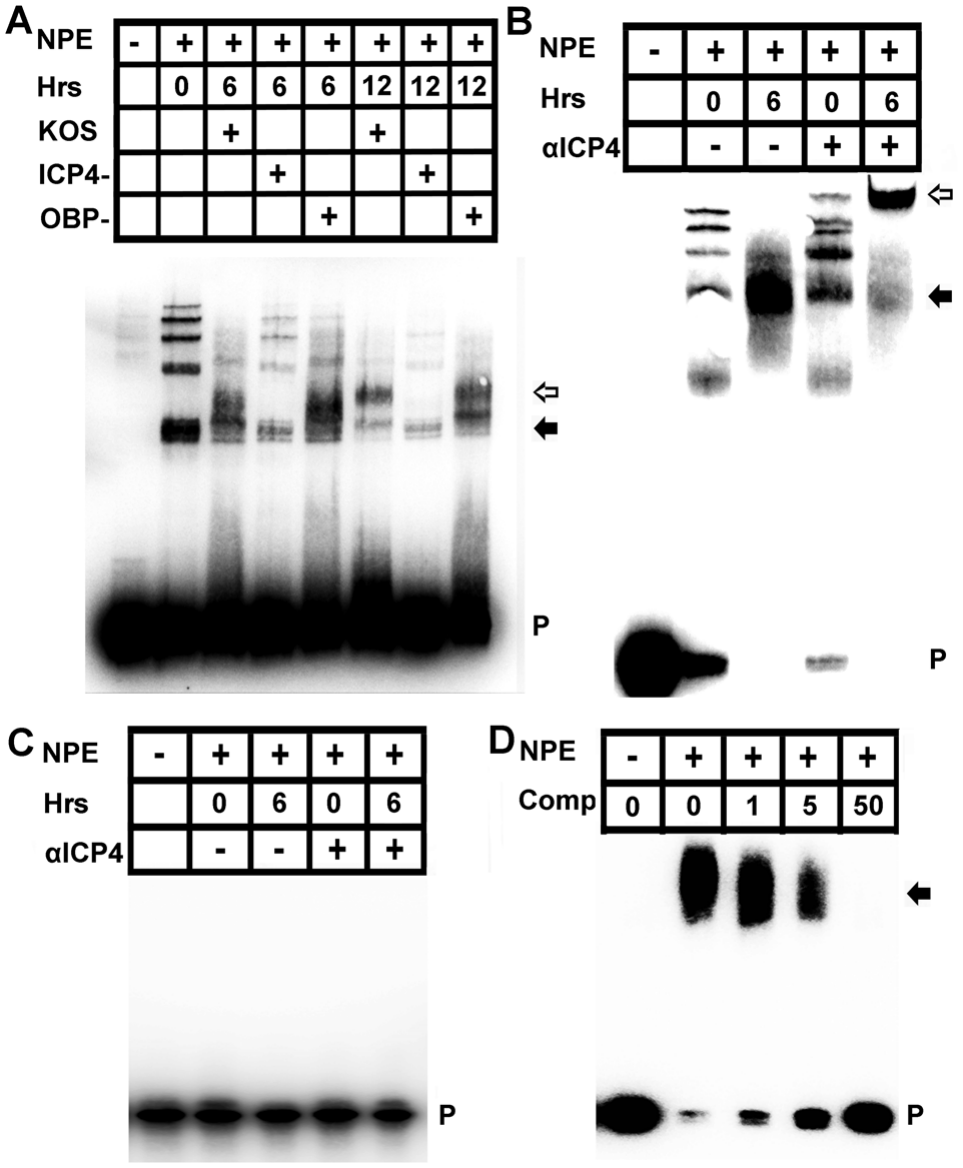
ICP4 binds the human VEGF-A promoter. (A) Image of EMSA using probe spanning base pairs −88 to +55 relative to the VEGF-A transcription start site with nuclear protein extracts (NPE) from 293 cells infected with HSV-1 KOS, HSV-1 KOS ICP4-, or the replication deficient control HSV-1 OBP-. Nuclear extracts were harvested at the indicated time PI with 3 pfu per cell of HSV-1. Infection with either HSV-1 KOS or HSV-1 OBP- led to changing EMSA shifts of VEGF-A probe, no shift was observed in cells infected with ICP4 deficient virus relative to uninfected control extracts. Free probe (P) indicated with a solid arrow denotes the probe and nuclear protein complex at 6 hours PI whereas the hollow arrow denotes the antibody shifted probe/nuclear protein complex at 12 hours PI. Representative of two experiments. (B) Supershift analysis was performed to determine if ICP4 bound −88/+55 probe from nuclear protein extracts of 293 cells infected with 3 pfu per cell HSV-1 KOS with or without antibody against ICP4 at 6 hour PI. The antibody further retarded the probe/nuclear protein complex; free probe (P) indicated by a solid arrow denotes the probe and nuclear protein complex whereas the hollow arrow denotes the antibody shifted probe/nuclear protein complex. (C) As a negative control for binding specificity, nuclear extracts were incubated with an oligo probe spanning an irrelevant region of the VEGF-A promoter (base pairs −1513 to −1338 relative to the transcription start site). Nuclear protein binding was not observed in EMSAs using UI or HSV-1 infected nuclear protein extracts incubated with either isotypic control or antibody against ICP4. (D) EMSA of −88/+55 VEGF-A probe incubated with HSV-1 infected nuclear protein extracts and increasing molar excesses of unlabeled oligonucleotide containing the ICP4 consensus site.

To test for a role for ICP4 in the complex binding the proximal VEGF-A promoter, nuclear extracts were harvested from 293 cells following infection with HSV-1 KOS, or ICP4^−^ null virus or OBP^−^ null virus. Infection with HSV-1 KOS or the OBP^−^ null virus led to a change in the EMSA shift of VEGF-A −88 to +55 probe within 6 hours PI that remained through 12 hours PI but no change in the probe shifts was observed in ICP4^−^ virus-infected nuclear extracts relative to uninfected extracts (Figure 7A). The ICP4 binding consensus DNA sequence A/GTCGTCNNNNYCGRC (N = any nucleotide, Y = pyrimidine, R = purine) is not present in the human VEGF-A promoter region [37], [38]. However, ICP4 undergoes extensive post-translational modifications that alter sequence affinity and ICP4 binds a wide variety of sequences with no apparent relation to its consensus sequence [37]–[40]. To determine if ICP4 bound the human VEGF-A promoter, −88 to +55 base pair probe was incubated with nuclear protein extracts harvested from cells at 6 hours PI and assayed for mobility supershift following addition of monoclonal antibody against HSV-1 ICP4. Addition of anti-ICP4 retarded probe/nuclear protein mobility establishing binding of ICP4 to the VEGF-A proximal promoter region (Figure 7B). ICP4 interaction with the proximal human VEGF-A promoter did not require GC box sequences from −85 to −52 bp relative to the transcription start site (Figure S2A). Furthermore, EMSA supershift analysis of probe spanning either −88 to −50 bp or probe spanning −50 to +55 bp indicated that the ICP4 binding site or sites were localized to −50 to +55 base pairs relative to the transcription start site (Figure S2B and C). ICP4 binding to the proximal human VEGF-A promoter was specific. Binding was not observed using oligo probe spanning an irrelevant sequence of the VEGF-A (bp −1513 to −1338 relative to the transcription start site, Figure 7C). In addition, nuclear protein complexes from HSV-1 infected cell extracts were also competitively disassociated from −88 to +55 bp probe by molar excesses of unlabeled oligo containing the ICP4 consensus sequence (Figure 7D).

**Figure 8:**
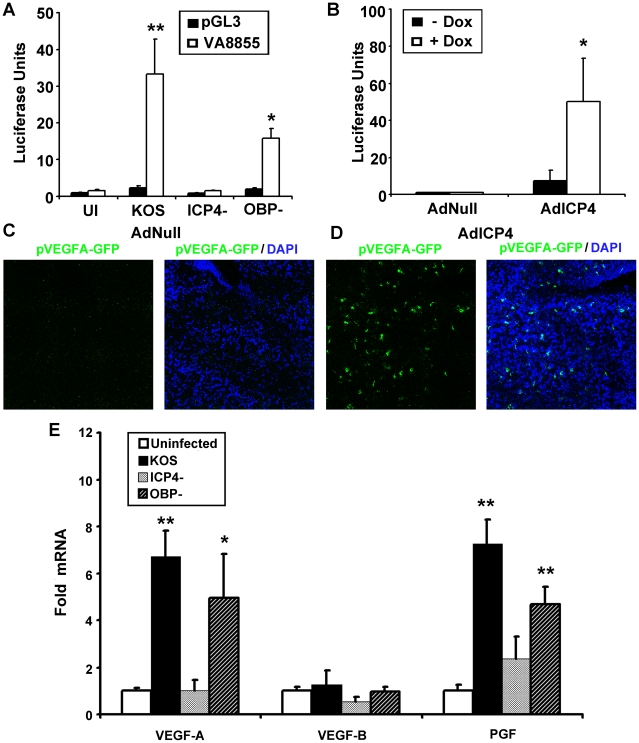
ICP4 expression is required and sufficient to drive VEGF-A transcription. (A) To determine if expression of ICP4 was required for activation of transcription at the VEGF-A promoter, human primary dermal keratinocytes were transfected with pGL3 or pVA8855 luciferase reporter were infected with 3 pfu per cell HSV-1 KOS, HSV-1 ICP4^−^ null virus, or HSV-1 OBP^−^ null virus and assayed for luciferase activity at 8 hours PI. (B) We tested to see if ICP4 expression was sufficent to drive transcription from the VEGF-A promoter by transfecting human primary dermal keratinocytes transfected with pVA8855 luciferase reporter. Transfected cells were then transduced with 10 pfu per cell of adenoviral vectors expressing the reverse tetracycline-regulated transactivator as well as either negative control vector (AdNull) or vector expressing TRE-driven ICP4 (AdICP4) and treated with either vehicle or vehicle plus 3 µM doxycycline. Luciferase activity was measured at 30 hours post-transduction. (C) The corneas of pVEGFA-GFP reporter mice were injected with a combination of either 10^5^ pfu AdNull and 10^4^ pfu AdrtTA or (D) 10^5^ pfu AdICP4 and 10^4^ pfu AdrtTA and treated with doxycycline via drinking water (2 mg/mL) for 5 days prior to examination via confocal microscopy for GFP reporter for VEGF-A (green, nuclei stained with DAPI shown in blue). (E) Transcript abundance of the VEGF family members; VEGF-A, VEGF-B, and PGF was measured by real-time RT-PCR in human primary keratinocytes at 8 hours PI with either HSV-1 KOS, ICP4-, or OBP- virus. VEGF-C and VEGF-D were not detected in any group. Abundance is expressed as fold induction relative to uninfected cells using the geometric mean of fold induction values from the housekeeping genes β-actin, TBP and PPIA. Statistical significance determination is also relative to uninfected cells. (** p<.01, * p<.05). Figures are representative of 2 experiments with an n = 4/group. Bars denote ± SEM.

We next sought to determine if ICP4 expression was sufficient to drive transcriptional enhancement of VEGF-A. As with 293 and THCE cells, luciferase reporter activity for the human proximal VEGF-A promoter is up-regulated in human primary dermal keratinocytes (HPKs) in an ICP4-dependent fashion (Figure 8A). To determine if ICP4 was sufficient for reporter expression, HPKs were transfected with either promoterless luciferase vector pGL3 or pVA8855 and transduced with adenoviral vector expressing the reverse tetracycline-controlled transactivator protein (AdrtTA) and either a negative control adenvoviral vector AdNull or vector expressing ICP4 under the control of a tetracycline response element (AdICP4). After incubation for 30 hours in the presence or absence of 3 µm doxycycline, cells were assayed for luciferase activity. The reporter was significantly up-regulated in AdICP4 transduced cells treated with doxycycline relative to AdICP4+ vehicle-treated and AdNull-treated controls (Figure 8B). We also tested ICP4 sufficiency for VEGF-A promoter activation in vivo by cornea stromal co-injection of either AdrtTA and AdNull or AdrtTA and AdICP4 into pVEGFA-GFP reporter mice. Mice were kept on doxycycline treated water (2 mg/mL) for 5 days before eyes were harvested and examined for GFP expression by confocal microscopy. The VEGFA-GFP reporter was not detectably induced in mouse corneas transduced with AdNull, but was abundantly expressed in AdICP4-transduced corneas (Figures 8C and D, respectively). Thus, ICP4 was required for transcriptional up-regulation of VEGF-A during HSV-1 infection and sufficient to augment transcription at the proximal human VEGF-A promoter.

Blockade of VEGF-A and its receptor VEGFR-2 are sufficient to block HSV-1- induced lymphangiogenesis but not angiogenesis [5], [12], [41], [42]. Lymphatic vessels typically express the VEGF family receptors VEGFR-2 and VEGFR-3 [9] while blood vessels express VEGFR-1 and VEGFR-2 [9], both of which bind VEGF-A [13]. We hypothesized that the mere partial dependence on blood vessel growth for VEGF-A may be due to the production of other VEGFR-1 ligands during HSV-1 infection. Transcript abundance for all five human VEGFs; VEGF-A, VEGF-B, VEGF-C, VEGF-D, and PGF was assayed by real-time RT-PCR in human primary keratinocytes infected with HSV-1 KOS, ICP4^−^ null, or OBP^−^ null virus. Transcripts encoding the VEGFR-3 ligands VEGF-C and VEGF-D were not detected by RT-PCR (Figure 8E). Likewise, the VEGFR-1 ligand VEGF-B was not up-regulated HSV-1 infection (Figure 8E). However, transcript for the VEGFR-1 ligands VEGF-A and PGF significantly increased following inoculation of human primary keratinocytes with HSV-1 KOS or OBP^−^ null virus (Figure 8E). As with VEGF-1 mRNA levels, increased transcript abundance of PGF mRNA was also ICP4-dependent (Figure 8E). At the time of writing it is unclear whether ICP4-dependent transcription of PGF occurs in the same direct fashion as with VEGF-A or whether ICP4 and HSV-1 indirectly activate transcription at the PGF promoter. However, our results indicate that HSV-1 infection induces mRNA transcription from cellular genes that encode two VEGFR1 ligands, and this induction is ICP4-dependent. Further testing will be required to determine if chemical or antibody-mediated blockade of VEGF-A and PGF ligands is sufficient to stop the angiogenic sequelae characteristic of ocular HSV-1 infection.

## Discussion

Despite the longstanding awareness of the impact of angiogenesis and contribution of VEGF-A during ocular HSV-1 infection [5], [41], [42], recognition of HSV-1 infected cells as a source of VEGF-A has occurred only recently [12]. Our study suggests that the preeminent source for VEGF-A during acute ocular infection is HSV-1 infected cells on the basis co-localization of VEGFA-GFP reporter with HSV-1 antigen (Figure 1, [12]) and experiments using HSV-1 infected cell-specific deletion of VEGF-A (Figure 2A). We had initially hypothesized that VEGF-A expression was driven by TLR or other pattern recognition receptors. TLR ligation drives expression of VEGF-A as well as other VEGF family members [16], [43], [44]. All TLRs signal through either MyD88 and/or TRIF [19]. Yet, MyD88 and TRIF deficiency had no impact on VEGF-A expression following HSV-1 inoculation. This result could be reconciled with functional redundancy between MyD88 and TRIF signaling TLRs allowing expression in the sole absence of either MyD88 or TRIF, but not in animals deficient in both genes. However, that appears unlikely as corneal infection with vesicular stomatitis virus does not drive detectable pVEGFA-GFP reporter expression in reporter mice [12] despite activation of MyD88- and TRIF-dependent TLRs [45], [46] as well as other pattern recognition receptors such as LRRFIP1 and RIG-I [14], [47], [48]. The possibility of TLR-driven VEGF-A expression during HSV-1 infection would also be particularly unlikely in human cells as the preeminent TLR mediating HSV-1 recognition in humans is TLR3 [49]. TLR3/TRIF signaling profoundly suppresses VEGF-A through interferon pathways [50] and is thus, unlikely to make a positive contribution during HSV-1 infection.

Conceivably non-TLR pattern recognition receptors could contribute to VEGF-A expression. However, ICP4- HSV-1 would be expected to activate similar pattern recognition receptors as parental HSV-1 yet did not up-regulate VEGF-A transcript. A null adenoviral vector also did not stimulate reporter for VEGF-A, while AdICP4 drove reporter expression in vitro and in vivo. Thus, two different model systems with considerable overlap for stimulation of pattern recognition receptors did not drive VEGF-A expression without concurrent expression of ICP4. In fact, innate pattern recognition may decrease VEGF-A expression. Viral pattern recognition receptors almost universally activate interferon pathways [51], [52], and interferon inhibits expression of VEGF-A [50].

Transcriptional up-regulation of VEGF-A was dependent on the HSV-1 transactivator ICP4. This observation and sequence similarities between the VEGF-A promoter and HSV-1 E gene promoters suggests that rather than being an innate response to viral infection, the HSV-1 transcriptional regulation program drives VEGF-A expression. VEGF-A mediates a number of responses that may be beneficial to the pathogen such as suppression of DC maturation [53] as well as growth and chemotaxis of neuronal axons [54], the ultimate target of HSV-1. All wild-type HSV-1 strains tested to date have maintained the ability to up-regulate VEGF-A transcript and promoter reporter. The highly homologous virus HSV-2, whose ICP4 is functionally interchangeable with HSV-1 ICP4 [55], also drove expression of VEGF-A. Shared expression of VEGF-A by these disparate viruses is suggestive of a natural selection mechanism. But activation of transcription of VEGF-A was maintained despite repeated passage within Vero cells, a fibroblast line expected to be deficient in the receptors for VEGF-A; VEGFR-1/2, though not specifically tested. In the absence of VEGFR-1/2 no conceivable survival benefits of VEGF-A expression would exist in culture, and a specifically evolved pathway would presumably be lost. Thus, rather than being an evolved pathway HSV-1 induced VEGF-A expression could be an incidental consequence of similarity between VEGF-A and HSV-1/2 promoters.

ICP4 bound the VEGF-A promoter independently of the three GC box sequences required for transcriptional up-regulation. In addition, we were not able to detect nuclear protein binding to these motifs in the VEGF-A promoter probe. We detected binding of nuclear proteins to probe containing a single isolated GC box (Figure S1). Therefore, we presume the lack of nuclear protein binding to GC boxes within VEGF-A promoter probe was due to the extensive secondary structure present within the VEGF-A promoter probe from −85 to −52 bp. The most obvious GC box binding candidate is the endogenous transcription factor Sp1 which is heavily phosphorylated during HSV-1 infection and plays a role in IE and E gene transcription [38], [56], [57]. However, it should be noted other members of the Sp transcription factor family also bind GC box sequences.

ICP4 bound within a region spanning bp −50 to +55 relative to the VEGF-A transcription start site (Figure S2). There are no sequences within this region with perfect homology to the ICP4 consensus binding sequence ATCGTCNNNNYCGRC (N = any nucleotide, Y = pyrimidine, R = purine, 12). This point is not a cause for concern as ICP4 affinity is degenerative with strong interactions involving sequences with no resemblance to the core consensus sequence [37]–[40], [58]. ICP4 and DNA interactions are also affected by oligomerization of ICP4 and extensive post-translation modification, at least some of which are dependent on additional HSV-1 proteins such as ICP27 [39]. As adenoviral expression of ICP4 was sufficient to drive transcription at the proximal VEGF-A promoter (Figure 7F–H). Therefore, it appears that post-translational modifications of ICP4 mediated by other HSV-1 proteins are not required for transcription initiation of VEGF-A. Instead, native ICP4 interaction with non-consensus elements or additional, possibly constitutively expressed DNA binding factors are sufficient to allow ICP4-dependent transcription of VEGF-A.

The effects of VEGF-A differ appreciably depending on isoform [7], [13], [59]. VEGF-A isoforms are generated by alternative splicing and vary in both extracellular matrix affinity and receptor signaling cascades elicited [7], [13]. HSV-1 ICP27 inhibits splicing [60] and may alter VEGF-A isoform expression from infected cells. VEGF-A expression during HSV-1 infection has been shown to drive both hem- and lymph-angiogenesis. The hemangiogenic consequences of VEGF-A expression are to be expected but the strongly pro-lymphangiogenic impact of HSV-1 elicited VEGF-A is at odds with studies showing only mild lymphodilation following expression of the most common VEGF-A isoforms [14]. VEGF-A expressed by HSV-1 infected cells may differ in either isoform or post-translation modifications and these differences may be responsible for disparate lymphangiogenic effects. Alternatively, expression of additional cytokines may alter the effects of VEGF-A. IL-1 and IL-6 promote angiogenesis during HSV-1 keratitis [42]. Moreover, we have identified another cytokine required along with VEGF-A for lymphangiogenesis during infection.

VEGF-A affects several systems that are not directly related to (lymph)angiogenesis. VEGF-A modifies dendritic cell function through ligation of DC expressed VEGFR-2, which inhibits maturation [53]. VEGF-A suppresses CCL21 expression by lymphatic endothelial cells, reducing DC migration to lymphatics [61], and ligation of neuronal- expressed VEGF receptors induces the growth of neuronal axons [54]. It remains to be seen whether HSV-1 elicited VEGF-A differs from naturally expressed forms with regards to suppression of DC maturation or neuronal axon growth.

In summary, our data provide conclusive evidence that HSV-1 drives expression of VEGF-A promoting angiogenic sequelae characteristic of ocular HSV-1 infection [8], [12], [41]. Not only was VEGF-A transcription dependent on the HSV-1 transactivator ICP4, the VEGFR-1 ligand PGF was also transcriptionally up-regulated in an ICP4- dependent fashion. The promoters between VEGF-A and PGF are highly homologous [62]. Future studies will examine the role of ICP4 in the expression of other PGF and the impact of ICP4 dependent expression of VEGFs on the host immune response, viral replication, and angiogenesis.

## Materials and Methods

### Ethics Statement

Animal treatment was consistent with the National Institutes of Health Guidelines n the Care and Use of Laboratory Animals. All experimental procedures were approved by the University of Oklahoma Health Sciences Center and Dean A. McGee Eye Institutes' Institutional Animal and Care Use Committee under the approved IACUC protocol number 10-024.

### Immunofluorescence Microscopy

Corneas were prepared and stained for specific antigens as previously described [63]. The sources of antibodies used are as follows; rabbit anti-HSV-1 and mouse anti-HSV-1 ICP4 (Abcam), goat anti-GFP (Serotec), Dylight 549 donkey anti-rabbit and FITC bovine anti-goat (Jackson ImmunoResearch). Images were taken using an Olympus IX81-FV500 epifluorescence/confocal laser-scanning microscope.

### Cytokine Measurement

Human and mouse VEGF-A levels were measured by cytokine bead array using a Bio-plex suspension array system (Bio-Rad) as previously described [64], with multiplex kits provided by Millipore.

### HSV-1 Infection and Mice

Male C57BL/6, B6129, MyD88−/− and TRIF−/− mice were purchased from Jackson Laboratories. Transgenic reporter mice expressing GFP under the proximal VEGF-A promoter were the generous gift of Dr. Brian Seed (Harvard University) and were constructed on an FVB background as previously described [65]. Mice with a floxed VEGF-A allele were generated as previously described [66]. A breeder pair was provided by Genentech. CD118−/− mice were maintained in the OUHSC barrier. Anesthetized mice were infected with HSV-1 by scarifying the cornea with a 25 gauge needle and applying 10^5^ pfu of HSV-1 per cornea. GFP reporter expression was visualized by secondary detection of GFP. Mice used were between 6 weeks and 6 months old and age matched controls were employed in each experiment.

### Cells and Virus

THCE cells were the generous gift of Dr. Jerry Shay (UT Southwestern) and were maintained in keratinocyte serum free media (Invitrogen) supplemented with 0.15 ng/ml EGF and 0.25 µg/ml bovine pituitary extract. Human 293 cells were maintained in DMEM (Gibco) supplemented with 10% FBS (Gibco). Primary human dermal keratinocytes (Lifeline Cell Technology) were maintained in complete Dermalife medium (Lifeline Cell Technology). HSV-1 virus stocks were propagated using Vero cells. The HSV-1 ICP4^−^ null virus, n12, is a nonsense mutant with a premature stop codon that only encodes the N-terminal 25% of the ICP4 protein [67] (generously provided by Dr. Neal Deluc, University of Pittsburgh). The HSV-1 OBP^−^ null virus, hr94, contains a lacZ reporter gene insertion that disrupts the origin binding protein (UL9) open-reading frame [59] (generously provided by Dr. Sandra Weller, University of Connecticut Health Sciences Center). The HSV-1 ICP0^−^ null virus, 7134, contains a lacZ open-reading grame exchanged in place of the ICP0 open-reading frame (generously provided by the late Dr. Priscilla Schaffer) [68]. HSV-1 d27-1 (ICP27^−^) null virus was a kind gift of Steve Rice (University of Minnesota Medical School, Minneapolis) [35]. HSV-1 expressing Cre recombinase under the ICP0 promoter was generated as described [69]. The parental strain HSV-1 SC16 was a gift from Dr. Weiming Yuan (University of Southern California, Los Angeles, CA). AdrtTA as well as AdNull and the TRE-regulated ICP4-expressing vector AdICP4 were constructed as previously described [68].

### Reporter Plasmids and Luciferase Assays

Luciferase reporter plasmids based on Promega's pGL3 luciferase reporter vector with luciferase driven by the sequences −2048/+50, −1290/+50, −790/+50, −415/+50, −268/+50, −85/+50, −52/+50 relative to the transcription start site of the human VEGF-A gene were constructed as previously described [70] and generously provided by Dr. Paul Fox (The Cleveland Clinic). For assays testing the importance of specific promoter elements within luciferase reporter plasmid −85/+50, a new vector driven by bp −88 to +55 of the human VEGF-A transcription start site was constructed as the original construction of −88/+50 removed the MCS site of pGL3, complicating further manipulations. The new vector, pVA8855, was created by amplifying the −88 to +55 base pair region of pLuc2098 using the primers.

VAProx8855-XhoI-5′-ACTGAACTCGAGCCCGGGGCGGGCCGGG-3′.

VAProx8855-HindIII-Rev-5′-TTCAGT AAGCTT CCCCCAGCGCCACGACCTCC-3′ and digesting the resulting PCR fragment and vector pGL3 in XhoI and HindIII (Promega) and ligated with T4 DNA ligase (Promega). The sequence integrity of the resulting plasmid was verified and luciferase expression following HSV-1 infection between pLuc135 and pVA8855 did not significantly differ. Site specific mutations of either EGR-1 consensus sequences or of the three GC boxes within pVA8855 were generated by PCR directed mutagenesis using Hotstart Turbo PFU DNA polymerase (Stratagene) and degradation of the original plasmid using Dpn I (Promega). The base sequence within pVA8855 corresponding to the region between −88 to −50 bp of the human VEGF-A promoter and sequences following mutation are listed below, all of which were verified by DNA sequencing.

pVA8855 (−88/−50) CCCGGGGCGGGCCGGGGGCGGGGTCCCGGCGGGGCGGAG

ΔEGR-1 CCCGGGGCGGGC**TA**GGGGCGGGGTCCC**TAA**GGGGCGGAG

ΔGC Box1 CCC**AACACA**GGCCGGGGGCGGGGTCCCGGCGGGGCGGAG

ΔGC Box2 CCCGGGGCGGGCCGG**AACACA**GGTCCCGGCGGGGCGGAG

ΔGC Box3 CCCGGGGCGGGCCGGGGGCGGGGTCCCGGC**AACACA**GAG

ΔGC Box1+2 CCC**AACACA**GGCCGG**AACACA**GGTCCCGGCGGGGCGGAG

ΔGC Box1+3 CCC**AACACA**GGCCGGGGGCGGGGTCCCGGC**AACACA**GAG

ΔGC Box2+3 CCCGGGGCGGGCCGG**AACACA**GGTCCCGGC**AACACA**GAG

ΔGC Box1+2+3 CCC**AACACA**GGCCGG**AACACA**GGTCCCGGC**AACACA**GAG

The primers used to generate above mutants are as follows; ΔEGR-1 For 5′-GCGGGCTAGGGGCGGGGTCCCTAAGGGGCGGAG-3′ Rev 5′-CTCCGCCCCTTAGGGACCCCGCCCCTAGCCCGC-3′

ΔGC Box1 For 5′-GGGCTCGAGCCCAACACAGGCCGGGGGCG-3′ Rev 5′-GCC CCCGGCCTGTGTTGGGCTCGAGCCCG-3′

ΔGC Box2 For 5′- GGGCGGGCGGAACACAGGTCCCGGCG-3′ Rev 5′-GCCGGGACCTGTGTTCCGGCCCGCCCCG-3′

ΔGC Box3 For 5′-GGTCCCGGC AACACAGAGCCATGCGCC-3′ Rev 5′-GGCGCA TGGCTCTGTGTTGCCGGGACC-3′

ΔGC Box1+2 For 5′-GGGCTCGAGCCCAACACAGGCCGGAACACAG-3′ Rev 5′-CTGTGTTCC GGCCTGTGTTGGGCTCGAGCCC-3′

Luciferase assays were performed by plating 25,000 293 cells or primary human dermal keratinocytes per well of a 96 well plate and transfecting cells the following day with 250 ng of plasmid per well and Lipofectamine 2000 (Invitrogen) per to the manufacturer's instruction. At 48 hours after transfection, transfected cells were infected with 3 pfu per cell of HSV-1 and luciferase activity was determined at the indicated times post infection relative to HSV-1 infection using Promega's firefly Luciferase Assay System per the manufacturer's instructions.

### Nuclear and Cytoplasmic Protein Extraction and EMSA

Nuclear and cytoplasmic extracts of 293 cells were harvested using NE-PER nuclear and cytplasmic protein extraction kit (Thermo Scientific). Protease activity was inhibited by addition of 1× Calbiochem Protease Inhibitor Cocktail. Protein concentration of the extracts was measured using BioRad BCA protein assay. The protein concentrations of nuclear and cytoplasmic extracts were normalized to 1000 µg/mL using NE-PER nuclear and cytoplasmic extraction buffers, respectively.

Oligonucleotides for EMSA were either chemically synthesized or generated by PCR amplification using Hotstart Turbo PFU DNA polymerase. Oligonucleotides of pVA8855, pVA8855 ΔEGR-1, pVA8855 ΔGC Box 1, pVA8855 ΔGC Box 2, pVA8855 ΔGC Box 3, pVA8855 ΔGC Boxes 1 and 2, pVA8855 ΔGC Boxes 1 and 3, pVA8855 ΔGC Boxes 2 and 3, pVA8855 ΔGC Boxes 1 and 2 and 3, were generated using primers biotinylated primers; biotin-5′-TAGCCCGGGCTCGAGCC-3′ and biotin-5′-GAATGCCAAGCTTCCCCCAG-3′ which amplifies a fragment corresponding to bp −88 to +55 relative to the transcription start site and biotin-5′-GCGGAGCCATGCGCCC-3′ and biotin-5′-GAATGCCAAGCTTCCCCCAG-3′ which amplifies a fragment corresponding to bp −50 to +55 relative to the transcription start site. Specific binding was verified by analyzing nuclear protein binding to biotinylated oligonucleotides spanning a sequence of the human VEGF-A promoter that was irrelevant to HSV-1 induced transcription of VEGF-A (−1513 to −1338 bp relative to the transcription start site), generated using the primers Biotin-5′-AGGCCTCAGAGCCCCAACTTTG-3′ and biotin-5′-CCTTACCTCCAAGCCCCCTTTTCC-3′. To analyze EMSA shifts corresponding to base pairs −88 to −50 bp of the human VEGF-A promoter, the wild-type oligonucleotide −88-50WT 5′-AGCCCGGGGCGGGCCGGGGGCGGGGTCCCGGCGGGG CGGAGCCAT-3′, or the GC box mutated corresponding oligonucleotide −88-50ΔGC 5′- AGCCCAACACAGGCCGGAACACAGGTCCCGGCAACACAGAGCCAT-3′. Binding of HSV-1 infected cell nuclear protein extracts was competitively inhibited using the ICP4 consensus containing oligonucleotide 5′-CAC TAT CGT CCA TAC CGA CCA CAC CGA CGA A-3′.

EMSAs were performed using Thermo Scientific's LightShift Chemiluminescent EMSA Kit according to the manufacturer's protocol with the exception that instead of the included EMSA binding buffer, the binding buffer used was composed as follows; 10 mM Tris-HCl, 5 mM MgCl_2_, 0.035% β-mercaptoethanol, 0.1% Triton X-100, and 2.5% glycerol (Sigma) at pH 7.5. Briefly, the procedure was as follows. Probe (20 femtomoles per 20 µL reaction volume) was incubated for 20 minutes in binding buffer with 2 µg of nuclear protein extract with or without 1.5 µL of anti-ICP4 antibody or isotypic control. Following binding, the reaction was electrophoresed in 0.5× TBE buffer in a 5% polyacrylamide gel (BioRad) using the BioRad Criterion cell system. Electrophoresed probe was transferred to a BioRad Zeta-probe nylon membrane and biotinylated oligonucleotide was detected via Streptavidin-horseradish peroxidase-mediated luminescence. Luminescence was detected with a FLUOstar Omega plate reader (BMG Labtech). Luminescence was normalized as luciferase units relative to the intensity of luminescence detected in non HSV-1 infected 293 cells transfected with the promoterless luciferase control vector pGL3.

### Real-time PCR

Corneas were harvested, and RNA was isolated at indicated time points PI using Trizol as per the manufacturer's instructions (Invitrogen). After RNA isolation, 2 µg RNA per sample was converted to cDNA using an RT system (Promega) using random primers according to the manufacturer's instruction. Samples were then analyzed via real-time PCR using Sybr Green supermix (Bio-Rad Laboratories) via an iCycler (Bio-Rad Laboratories) as previously described [71]. The abundance of VEGF-A/C/D cDNA relative to the housekeeping gene β-actin was calculated as 2^−ΔΔCt^. For analysis of VEGF-A up-regulation in THCE cells, VEGF-A fold induction was determined using a panel of three housekeeping genes to control for effects of HSV-1 on housekeeping gene expression. VEGF-A, B, C, D, or PGF cDNA relative to β-actin, TBP, or PPIA was calculated as described for β-actin, and the geometric mean of the threefold induction values was taken as being the individual fold induction for the respective sample. VEGF-C and VEGF-D were not detected in human 293 cells or HPKs in our hands. The validity of these primers has already been verified previously by other groups [72], [73]. THCE cells were plated at densities of 3×10^5^ cells per well in 12-well plates and then infected with 3 pfu per cell HSV-1 the next day before harvesting RNA at the indicated time point using Trizol. Primers were purchased from Sigma-Aldrich and sequences used were as follows: Mu VEGF-A forward, 5′-CTGCTGTACCTCCACCATGC-3′; Mu VEGF-A reverse, 5′-TCACTTCATGGGACTTCTGCTCT-3′; Mu VEGF-C forward, 5′-CTGfGGAAATGTGCCTGTGAATG-3′; Mu VEGF-C reverse, 5′-ATTCGCACACGGTCTTCTGTAAC-3′; Mu VEGF-D forward, 5′-CAAGACGAGACTCCACTGCC-3′; Mu VEGF-D reverse, 5′-GCACTCACAGCGATCTTCATC-3′; Mu β-actin forward, 5′-CTTCTACAATGAGCTGCGTGTG-3′; Mu β-actin reverse, 5′-TTGAAGGTCTCAAACATGATCTGG-3′; Hu VEGF-A forward, 5′-AGGAGGAGGGCAGAATCATCA-3′; Hu VEGF-A reverse, 5′-CTCATTGGATGGCAGTAGCT-3′; Hu VEGF-B forward, 5′-TCGCCGCACTCCTGCAGCTG-3′; Hu VEGF-B reverse 5′-CGAGTATACACATCTATCCATGACAC-3′; Hu VEGF-C forward 5′-TCAAGGACAGAAGAGACTATAAAATTTGC-3′ validated in reference 72; Hu VEGF-C reverse 5′-ACTCCAAACTCCTTCCCCACAT-3′; Hu VEGF-D forward 5′-ATGGACCAGTGAAGCGATCAT-3′, validated in reference 78; Hu VEGF-D reverse 5′-CAGCTTCCAGTCCTCCAGAGTGA-3′; Hu PGF forward 5′-GGAACGGCTCGTCAGAGGTG-3′; Hu PGF reverse 5′-CGACGTCCACCAGCCTCTC-3′; Hu β-actin forward, 5′-AGCCTCGCCTTTGCCGA-3′; Hu β-actin reverse, 5′-CATGTCGTCCCAGTTGGTGAC-3′; Hu TBP forward, 5′-TGCACAGGAGCCAAGAGTGAA-3′; Hu TBP reverse, 5′-CACATCACAGCTCCCCACCA-3′; Hu PPIA forward, 5′-GTCAACCCCACCGTGTTCTT-3′, PPIA reverse, 5′-CTGCTGTCTTTGGGACCTTGT-3′, Hu EGR-1 For 5′-CAGCCCTACGAGCACCTGACC-3′, Hu EGR-1 Rev 5′-GAGTGGTTTGGCTGGGGTAAC-3′.

For assays of VEGF-A transcript following infection with HSV-1 and treatment with specific inhibitors, the following concentrations were used throughout the 12 hour course of the assay; cycloheximide (Sigma) 100 µg/mL, acyclovir (Sigma) 200 µM, and U0126, SB206580, and SP600125 (all purchased from Sigma) were used at concentrations of 10 µM.

### Reduction of EGR-1 via siRNA

To reduce EGR-1, 293 cells were plated in 24 plates at a density of 10^5^ cells per well in DMEM without antibiotics. Cells were treated with either siPort Amine transfection reagent, or with transfection reagent and either negative control siRNA (4390843 Ambion), or siRNAs against EGR-1 (4538 and 4539 Ambion) with siRNAs applied at a final concentration of 20 nM in serum free Optimem (Invitrogen). After a 24 hour incubation, medium was removed and replaced with DMEM without antibiotics, and the plate was used after an additional 24 hour incubation. Reduction of EGR-1 was verified by real time RT-PCR to be greater that 60% in both 4538 and 4539 uninfected groups relative to negative siRNA controls and EGR-1 transcript remainded below levels in negative control-treated uninfected cells even 12 post HSV-1 infection.

### Statistics

Comparisons between multiple treatment groups were performed using one-way analysis of variance and Tukey's multiple comparison tests. All statistical analysis was performed with GBSTAT (Dynamic Micro Systems).

## Supporting Information

Figure S1 **GC boxes and nuclear protein binding.** (A) Diagram of EMSA probe containing base pairs −88 to −50 of the human VEGF-A promoter (−88-50WT) or a GC box mutated derivative (−88-50ΔGC) used to detect GC box dependent nuclear protein binding. (B) EMSA image of either wild-type or GC box mutated probe incubated with 0, 6, or 12 hour PI nuclear protein extracts harvested from 293 cells infected with 3 pfu per cell of HSV-1 McKrae. (C) Despite not observing GC box dependent binding to −88-50 wild type probe, a probe containing a single, isolated GC box bound nuclear proteins in extracts harvested from 293 cells at 0, 6, and 12 hours PI with 3 pfu per cell HSV-1 McKrae. Figures B and C are representative of 3 experiments.
(TIF)

Figure S2 **GC boxes are not required for ICP4 binding.** (A) EMSA using nuclear proteins extracts harvested from 293 cells at 6 hours PI with 3 pfu per cell HSV-1 McKrae incubated with either wild type −88 to +55 bp probe (pVA8855) or GC box mutated probe (ΔGC123) and either isotypic control or antibody against ICP4. (B) Diagram of probes spanning the proximal human VEGF-A promoter used determine the ICP4 binding region. (C) EMSA images using 293 nuclear protein extracts at 6 hours PI with either −88 to −50 base pair probe or −50 to +55 base pair probe with either isotypic control or antibody against ICP4. Free probe (P) indicated by a solid arrow denotes native probe/nuclear protein complex whereas the hollow arrow denotes antibody supershifted complex. EMSA images are representative of 3 experiments.
(TIF)

Figure S3 **ICP27 is not required for activation of transcription at the VEGF-A promoter.** (A) Immunofluorescence images of 293 cells stained with anti ICP4 monoclonal antibody or control IgG (red) at the indicated time PI with HSV-1 KOS or ICP27 deleted virus (nuclei stained with DAPI shown in blue). (B) ICP4 staining (red) of human 293 cells at 12 hours PI with either HSV-1 KOS or ICP27- virus. Nuclei are denoted by dashed white lines. (C) Human 293 cells were transfected with pVA8855 luciferase reporter plasmid for 48 hours, and were assayed for luciferase activity at the indicated times PI with 3 pfu per cell of HSV-1 KOS, ICP4^−^ null, or ICP27^−^ null virus. Luciferase activity was normalized to luminescence values of uninfected human 293 cells transfected with pGL3. Representative of two experiments (**, p<0.01). Bars denote ± SEM.
(TIF)
