# Supplementary figures and images for: The Herpes Simplex Virus-1 Transactivator Infected Cell Protein-4 Drives VEGF-A Dependent Neovascularization
Source: PLoS Pathog. 2011 Oct 6;7(10):e1002278. doi: 10.1371/journal.ppat.1002278 (PMC3188529; doi:10.1371/journal.ppat.1002278)

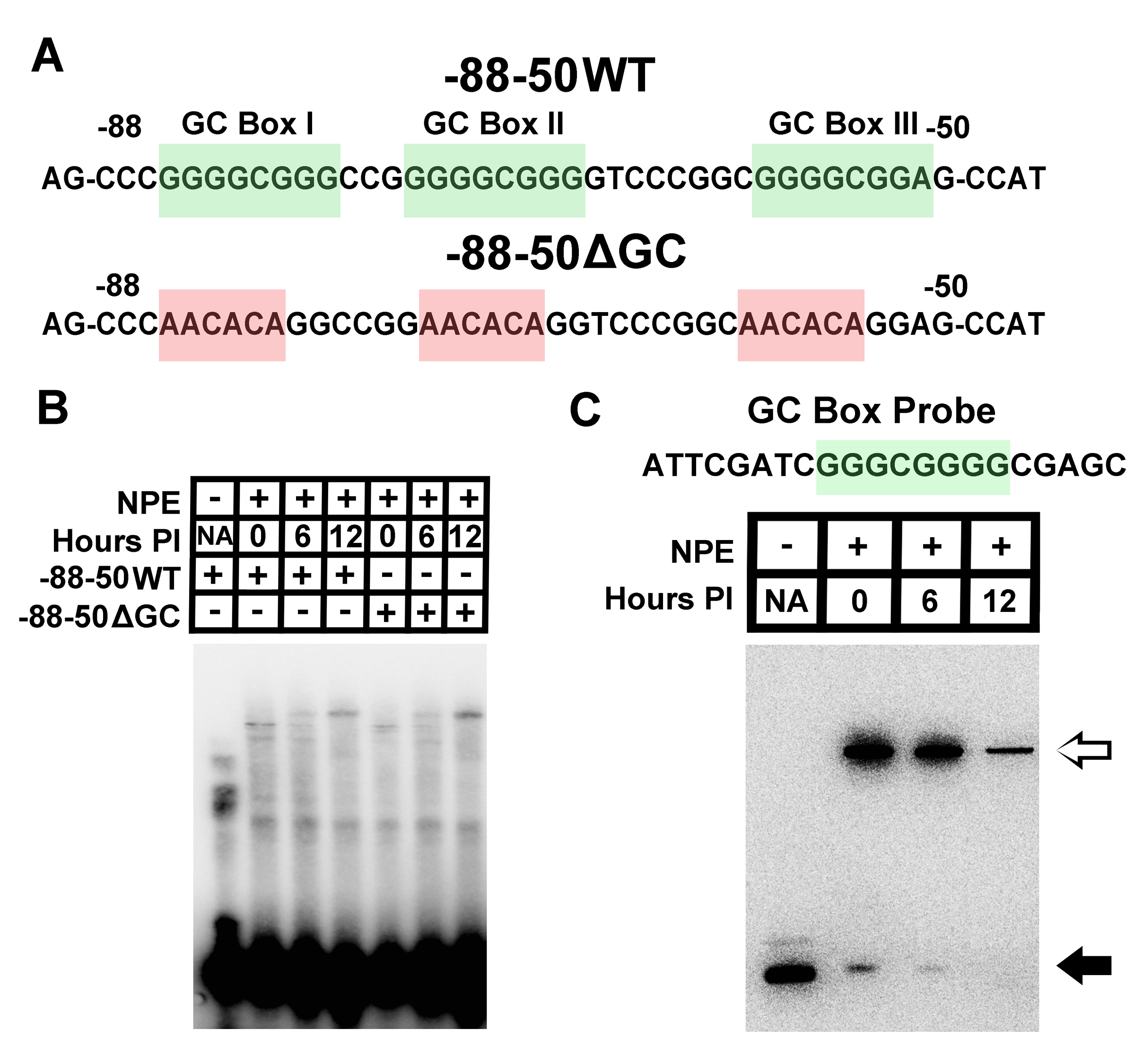

Supplement: Figure S1 — GC boxes and nuclear protein binding. (A) Diagram of EMSA probe containing base pairs −88 to −50 of the human VEGF-A promoter (−88-50WT) or a GC box mutated derivative (−88-50ΔGC) used to detect GC box dependent nuclear protein binding. (B) EMSA image of either wild-type or GC box mutated probe incubated with 0, 6, or 12 hour PI nuclear protein extracts harvested from 293 cells infected with 3 pfu per cell of HSV-1 McKrae. (C) Despite not observing GC box dependent binding to −88-50 wild type probe, a probe containing a single, isolated GC box bound nuclear proteins in extracts harvested from 293 cells at 0, 6, and 12 hours PI with 3 pfu per cell HSV-1 McKrae. Figures B and C are representative of 3 experiments. (TIF) [file ppat.1002278.s001.tif]

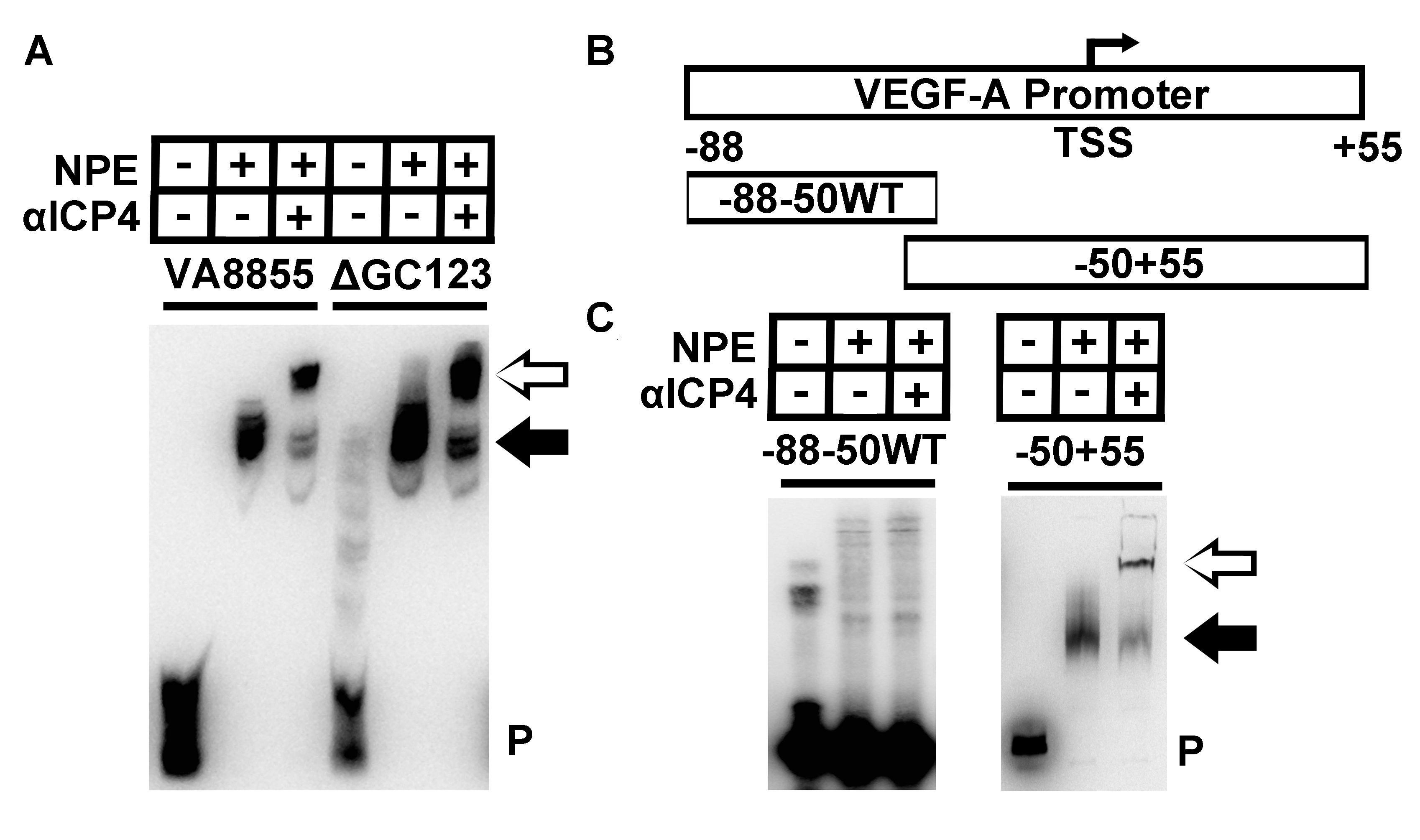

Supplement: Figure S2 — GC boxes are not required for ICP4 binding. (A) EMSA using nuclear proteins extracts harvested from 293 cells at 6 hours PI with 3 pfu per cell HSV-1 McKrae incubated with either wild type −88 to +55 bp probe (pVA8855) or GC box mutated probe (ΔGC123) and either isotypic control or antibody against ICP4. (B) Diagram of probes spanning the proximal human VEGF-A promoter used determine the ICP4 binding region. (C) EMSA images using 293 nuclear protein extracts at 6 hours PI with either −88 to −50 base pair probe or −50 to +55 base pair probe with either isotypic control or antibody against ICP4. Free probe (P) indicated by a solid arrow denotes native probe/nuclear protein complex whereas the hollow arrow denotes antibody supershifted complex. EMSA images are representative of 3 experiments. (TIF) [file ppat.1002278.s002.tif]

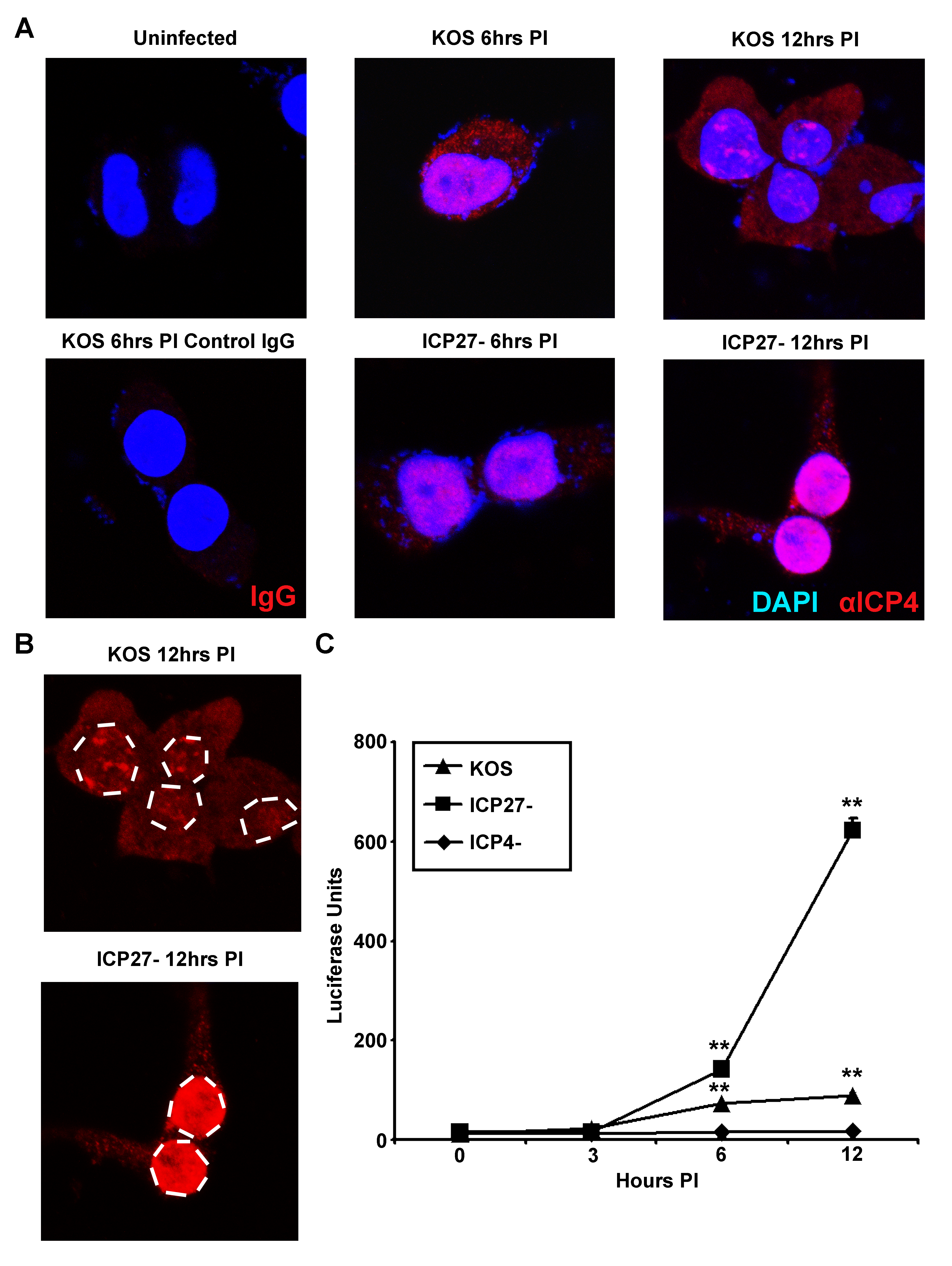

Supplement: Figure S3 — ICP27 is not required for activation of transcription at the VEGF-A promoter. (A) Immunofluorescence images of 293 cells stained with anti ICP4 monoclonal antibody or control IgG (red) at the indicated time PI with HSV-1 KOS or ICP27 deleted virus (nuclei stained with DAPI shown in blue). (B) ICP4 staining (red) of human 293 cells at 12 hours PI with either HSV-1 KOS or ICP27- virus. Nuclei are denoted by dashed white lines. (C) Human 293 cells were transfected with pVA8855 luciferase reporter plasmid for 48 hours, and were assayed for luciferase activity at the indicated times PI with 3 pfu per cell of HSV-1 KOS, ICP4− null, or ICP27− null virus. Luciferase activity was normalized to luminescence values of uninfected human 293 cells transfected with pGL3. Representative of two experiments (**, p<0.01). Bars denote ± SEM. (TIF) [file ppat.1002278.s003.tif]
